# Hierarchy and interconnected networks in the WhiB7 mediated transcriptional response to antibiotic stress in *Mycobacterium abscessus*

**DOI:** 10.1371/journal.pgen.1011060

**Published:** 2023-12-06

**Authors:** Kelley Hurst-Hess, Charity McManaman, Yong Yang, Shamba Gupta, Pallavi Ghosh

**Affiliations:** 1 Division of Genetics, Wadsworth Center, New York State Department of Health, Albany, New York, United States of America; 2 School of Public Health, University at Albany, Albany, New York, United States of America; Colorado State University, UNITED STATES

## Abstract

*Mycobacterium abscessus* is intrinsically resistant to antibiotics effective against other pathogenic mycobacteria largely due to the drug-induced expression of genes that confer resistance. WhiB7 is a major hub controlling the induction of resistance to ribosome-targeting antibiotics. It activates the expression of >100 genes, 7 of which are known determinants of drug resistance; the function of most genes within the regulon is however unknown, but some conceivably encode additional mechanisms of resistance. Furthermore, the hierarchy of gene expression within the regulon, if any, is poorly understood. In the present work we have identified 56 WhiB7 binding sites using chromatin immunoprecipitation sequencing (CHIP-Seq) which accounts for the WhiB7-dependent upregulation of 72 genes, and find that *M*. *abscessus* WhiB7 functions exclusively as a transcriptional activator at promoters recognized by σ^A^/σ^B^. We have investigated the role of 18 WhiB7 regulated genes in drug resistance. Our results suggest that while some genes within the regulon (eg. *erm41*, *hflX*, *eis2* and the ABCFs) play a major role in resistance, others make smaller contributions (eg. MAB_4324c and MAB_1409c) and the observed hypersensitivity ΔMab*whiB7* is a cumulative effect of these individual contributions. Moreover, our CHIP-Seq data implicate additional roles of WhiB7 induced genes beyond antibiotic resistance. Finally, we identify a σ^H^-dependent network in aminoglycoside and tigecycline resistance which is induced upon drug exposure and is further activated by WhiB7 demonstrating the existence of a crosstalk between components of the WhiB7-dependent and -independent circuits.

## Author summary

The induction of multiple genes that confer resistance to structurally diverse ribosome-targeting antibiotics is funneled through the induction of a single transcriptional activator, WhiB7, by antibiotic- stalled ribosomes. This poses a severe restriction in *M*. *abscessus* therapy as treatment with one ribosome-targeting antibiotic confers resistance to all other ribosome-targeting antibiotics. Here we uncover the intricacies of the WhiB7 regulatory circuit, identify three previously unknown determinants of aminoglycoside resistance and unveil a communication between WhiB7 dependent and independent components. This not only expands our understanding of the antibiotic resistance potential of *M*. *abscessus* but can also inform the development of much needed therapeutic options.

## Introduction

The genus *Mycobacterium* is comprised of obligate pathogens and environmental bacteria that are either saprophytes or opportunistic pathogens, and are all known to demonstrate varying levels of intrinsic antibiotic resistance that restricts therapeutic options against their infections [1–4]. This innate insusceptibility is attributed to a combination of a complex cell wall that poses a permeability barrier, and the expression of chromosomally encoded effectors that confer resistance [1–4]. Antibiotic resistance effectors include efflux pumps as well as enzymes that either modify/inactivate the target or the drug; these can be constitutively expressed but are more frequently induced as a consequence of the transcriptional reprogramming that occurs upon drug exposure. Within the genus, *Mycobacterium abscessus* is an exceptionally drug-resistant, rapidly-growing, non-tuberculous mycobacterium (NTM) causing acute and chronic pulmonary disease in patients with underlying lung damage such as cystic fibrosis (CF) and COPD, as well as skin and soft tissue infections post-surgery/-trauma [5–8]. The multi-drug resistance phenotype of *M*. *abscessus* presents a major challenge in treatment of its infections [7,9]. Therapy involves a combination of a macrolide (clarithromycin or azithromycin) and intravenous amikacin and cefoxitin/imipenem for ~12–18 months with an average clearance rate of ~45% [10]. These dismal eradication rates are attributed, at least in part, to antibiotic induced induction of resistance determinants that limit the efficacy of the regimen [11–13].

The best studied master-regulator of the antibiotic-induced reprogramming in mycobacteria is WhiB7 which belongs to the WhiB-like (Wbl) family of transcriptional regulators found exclusively in actinomycetes [14]. It is characterized by the presence of four invariant cysteine residues that bind an [4Fe-4S] cluster, a conserved β-turn G[I/V/L]W[G/A]G) motif present in all Wbl proteins, a WhiB7 specific (EPW) motif adjacent to the β-turn and a C-terminal DNA binding AT-hook (RGRP) [14,15]. *whiB7* is one of the earliest genes induced to varying extents when mycobacteria are exposed to subinhibitory concentrations of structurally unrelated antibiotics that target both the 50S and 30S subunits of the ribosome [12,16–18]. A deletion of *whiB7* in *M*. *abscessus*, *M*. *smegmatis* and *M*. *tuberculosis* results in prominent hypersensitivity to macrolide, lincosamide, chloramphenicol and the aminoglycosides amikacin (AMK) and spectinomycin (SPC), moderate hypersensitivity to tigecycline (TIG), linezolid (LZD) and the aminoglycoside, streptomycin (STR), and mild sensitivity to the aminoglycoside, apramycin (APR) [12,17]. Although subinhibitory levels of tetracycline (TET) strongly induces *whiB7* in *M*. *abscessus*, the expression of the TET resistance determinant is WhiB7 independent [19]. In addition to antibiotics, *whiB7* expression is induced by compounds that perturb respiration and redox balance, and physiological stresses such as heat shock, iron starvation and entry into stationary phase [20,21]. Finally, *whiB7* induction has also been observed upon infection of mycobacteria into macrophages and in lungs of infected mice suggesting its importance in virulence [22,23].

The WhiB7 genes in actinobacteria are typically preceded by long leader sequences that include an upstream ORF (uORF) and a rho-independent terminator that functions as an attenuator [24,25]. Ribosome stalling in the presence of antibiotics leads to formation of an anti-terminator hairpin utilizing overlapping sequences in the uORF and the terminator leading to suppression of termination and induction of the downstream *whiB7* gene [24,25]. The WhiB7 protein then functions as a transcriptional activator and regulates the expression of >100 genes that comprise the WhiB7 regulon [12,17]. Seven genes within this regulon are primary determinants of macrolide- lincosamide resistance (*erm41*, *hflX*, MAB_2355c and MAB_1846), SPC resistance (MAB_2780c), AMK resistance (MAB_4532c) and kanamycin resistance (MAB_4395) [12,17,26–30]. However, the role of the vast majority of genes within the regulon, the WhiB7 dependent effectors of STR, TIG and APR resistance, as well as the hierarchy and the molecular networks, if any, are poorly understood in mycobacteria. In the present work we have determined the direct targets of *M*. *abscessus* WhiB7 using chromatin immunoprecipitation sequencing (CHIP-Seq) and investigate the role of 18 direct targets of WhiB7 in drug resistance. Finally, we also elucidate a drug-induced σ^H^-dependent network involved in aminoglycoside and tigecycline resistance that intersects with the WhiB7 dependent pathway.

## Results

### WhiB7-dependent transcriptional reprogramming in *M*. *abscessus* upon antibiotic exposure

**Fig 1:**
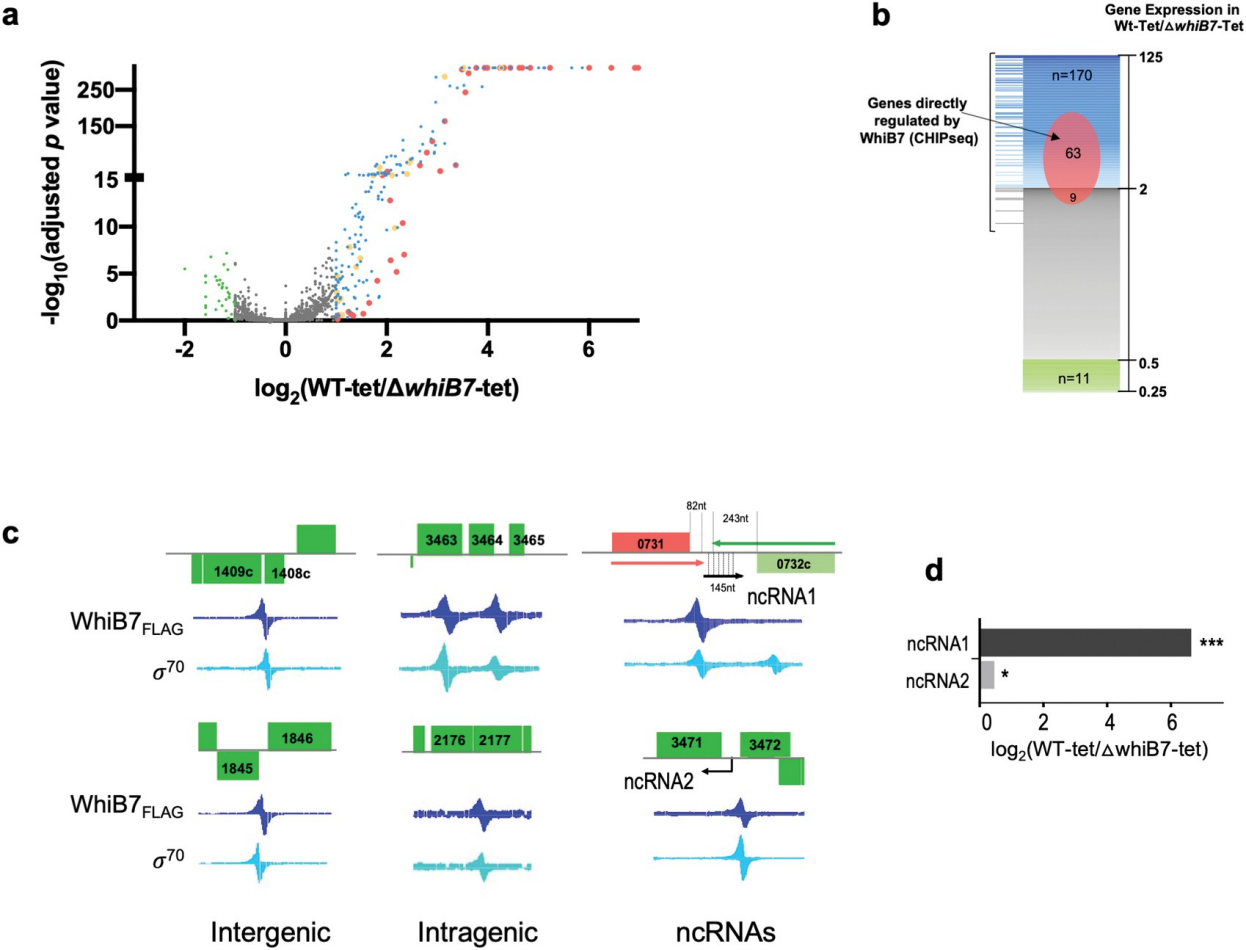
Determination of the *M*. *abscessus* WhiB7 dependent regulon and WhiB7 binding sites. **a)** Volcano plot of differentially expressed genes in *M*. *abscessus* ATCC19977 wild-type and Δ*MabwhiB7* strains upon exposure to TET (16μg/mL for 45 mins) determined by RNASeq. Four biological replicates were used of each sample. Genes differentially upregulated >2-fold in wild-type are indicated in blue, red and orange, and genes differentially downregulated >2-fold in wild-type are indicated in green. The red circles correspond to genes immediately adjacent to WhiB7 binding sites and orange circles correspond to operonic genes. **b)** Graphical representation of the WhiB7 regulon showing proportion of genes identified using CHIP-Seq. Location of direct targets of WhiB7 shows the fold downregulation of each target in *ΔMabwhiB7* **c)** Representative examples of WhiB7 binding sites at intergenic locations, within genes and transcribing ncRNAs visualized on Signal Map are shown. **d)** WhiB7 dependent induction of ncRNAs in wild-type and Δ*MabwhiB7* strains upon exposure to TET (16μg/mL for 45 mins). Region of complementarity between ncRNA1 and MAB_0732c transcript is shown using dashed vertical lines. p_*adj*_ *=* 0 (***) and <0.01 (*).

In a previous study we compared the changes in gene expression of ΔMab*whiB7* and ΔMab*whiB7*::*phspMabwhiB7* strains in the absence of antibiotic using RNA sequencing (RNAseq) and identified 229 genes comprising the *whiB7* responsive regulon in *M*. *abscessus* using the criteria of >2-fold induction in the *whiB7* overexpressing strain as compared to the Δ*MabwhiB7* mutant (*p*_*adj*_ <0.01) [12]. To identify genes that are dependent upon WhiB7 for expression upon antibiotic exposure, we followed the global changes in gene expression in wild-type *M*. *abscessus* and a Δ*MabwhiB7* strain. The elucidation of gene expression changes in Δ*MabwhiB7* was facilitated by the fact that TET strongly induces *whiB7* expression; however, Δ*MabwhiB7* is not TET susceptible since the determinants of TET resistance lie outside the WhiB7 regulon. This enabled us to determine the changes in gene expression in wild-type and Δ*MabwhiB7* backgrounds under identical antibiotic exposure conditions. Upon treatment of the strains with 16μg/ml TET (1XMIC) for 45 mins, we identified 181 genes that were differentially regulated >2-fold (*p*_*adj*_ <0.05) and were largely concordant with the previously reported WhiB7 responsive dataset. Of the 181 genes, the expression of 170 genes was reduced >2-fold in Δ*MabwhiB7* compared to wild-type bacteria, while the expression of 11 genes increased >2-fold in the mutant. (Fig 1A and 1B and S1 Data).

### Identification of chromosomal binding sites of MabWhiB7 using CHIP-Seq

The 181 genes comprising the MabWhiB7 regulon presumably include both direct and indirect targets of WhiB7. To identify the genes that are directly regulated by WhiB7 we determined the genome-wide binding profile of MabWhiB7 using CHIP-Seq. For this purpose we constructed 3 strains: i) the Mab*whiB7* gene was 3X-FLAG tagged at the 3’-end, integrated at the L5 attachment site of a Δ*MabwhiB7* strain and expressed using its native promoter and upstream regulatory elements contained within a 650 nt leader sequence (Δ*whiB7*::*p*_*nat*_*whiB7*_***C-FLAG***_), ii) the Mab*whiB7* gene was 3X-FLAG tagged at the 3’-end, integrated at the L5 attachment site of a Δ*MabwhiB7* strain and constitutively expressed (Δ*whiB7*::*p*_*hsp*_*whiB7*_***C-FLAG***_) iii) Mab*whiB7* gene was 3X-FLAG tagged at the 5’-end and constitutively expressed from a chromosomal location of a Δ*MabwhiB7* strain (Δ*whiB7*::*p*_*hsp*_*whiB7*_***N-FLAG***_). Control experiments showed that addition of a C-terminal or N-terminal FLAG-tag did not interfere with WhiB7 function (S1 Fig). The strains were grown to mid-exponential phase (OD_600_ = 0.6); Δ*whiB7*::*p*_*nat*_*whiB7*_*C-FLAG*_ was further treated with 16μg/ml TET; DNA-nucleoprotein complexes were immunoprecipitated with anti-FLAG or anti-σ^70^ monoclonal antibodies followed by library preparation and Illumina sequencing. Using a previously published Python script, Peakcaller [31,32], we identified a total of 56 WhiB7 binding sites across all 3 strains (Table 1) of which, 51 were located between genes that were transcribed either divergently or in the same direction; 5 peaks were found to be intragenic (Fig 1C and Table 1). Furthermore, all WhiB7 binding sites were also associated with a σ^70^ peak that recognizes both Group I and II sigma factors, implying that WhiB7 can affect transcription exclusively at promoters transcribed by σ^A^/σ^B^ [32] (S2 Table).

**Table 1:** CHIPseq of MabWhiB7 binding sites. Genetic location and coordinates of peaks, gene function and fold downregulation in ΔMabwhiB7 (RNAseq) are shown. Identity of gene regulated by WhiB7 binding from MEME analysis is also included. Strain in which each peak was identified is also noted. Strain 1: Δ*whiB7*::*p*_*nat*_*whiB7*_***3’-FLAG***_, Strain 2: Δ*whiB7*::*p*_*hsp*_*whiB7*_***3’-FLAG***_ and Strain 3: Δ*whiB7*::*p*_*hsp*_*whiB7*_***5’-FLAG***_.

Peak #	WhiB7 CHIPseq peak			Gene Regulated	Gene names	Gene description	Fold downregulation in △whiB7-Tet (RNAseq)	Strain where detected
	Coordinates	FAT score	Genetic location					
1	3842377	1	MAB_3786c/ MAB_3787	MAB_3786c		Hypothetical protein	125.79	1,2,3
2	2411990	2	MAB_2355c/MAB_2356	MAB_2355c		Putative ABC transporter ATP-binding protein	120.42	1,2,3
3	4615555	2	MAB_4532c/MAB_4533	MAB_4532c	*eis2*	Acetyltransferase	86.89	1,2,3
4	3011060	1	upstream of MAB_2956	MAB_2956		hypothetical protein—GATB Yqey superfamily	64.05	1,2,3
5	1136821	1	upstream of MAB_1125c	MAB_1125c		GNAT acetyltransferase	38.44	1,2,3
6	4404776	1	upstream of MAB_4324c	MAB_4324c		Putative GNAT acetyltransferase, RimI superfamily	37.53	1,2,3
7	4370213	1	upstream of MAB_4294	MAB_4294		Probable aspartate aminotransferase	28.58	1,2,3
8	158505	1	MAB_0163c/MAB_0164	MAB_0163c		Aminoglycoside phosphotransferase	25.62	1,2,3
9	3095974	1	upstream of MAB_3042c	MAB_3042c	*hflX*	HflX	24.65	1,2,3
10	2893879	1	MAB_2844c/MAB_2845	MAB_2845		aconitate methyltransferase	21.84	1,2,3
11	2954769	2	upstream of MAB_2903	MAB_2903		Hypothetical protein	21.77	1,2,3
12	2828542	1	upstream of MAB_2780c	MAB_2780c		MFS transporter	19.90	1,2,3
13	1346473	1	MAB_1344c/MAB_1345	MAB_1344c		Putative glucose-4,6-dehydratase/ oxidoreductase	18.80	1,3
14	2345935	1	upstream of MAB_2297	MAB_2297	*erm41*	erm41	15.90	1,2,3
15	1413707	11	upstream of MAB_1409c	MAB_1409c		Putative drug antiporter protein precursor (MFS)	15.30	1,2,3
16	4707211	1	upstream of MAB_4621c	MAB_4621c		Putative GNAT acetyltransferase, Rim L superfamily	13.64	1,2,3
17	3966090	1	upstream of MAB_3913	MAB_3913		Putative translocator (RhtB family- efflux?)	12.27	1,2,3
18	1299192	1	MAB_1295c/MAB_1296	MAB_1296		Hypothetical protein	11.75	1,3
19	1341921	1	upstream of MAB_1340	MAB_1340		nucleoride binding protein (YgdF family)	11.23	1,2,3
20	3507827	4	upstream of MAB_3465	MAB_3465		Putative sulfate transporter/antisigma-factor antagonist	8.33	1,2,3
21	874984	1	upstream of MAB_0880	MAB_0880		MFS transporter	6.94	1,2,3
22	405480	33	upstream of MAB_0404c	MAB_0404c		Putative GNAT acetyltransferase	6.31	1,2,3
23	2944991	1	upstream of MAB_2892c	MAB_2892c	*pdxS*	PdxS pyridoxal biosynthesis lyase	5.08	1,2,3
24	1844715	11	MAB_1845c/MAB_1846	MAB_1846		Putative ABC transporter ATP-binding protein	4.57	1,2,3
25	5030041	1	MAB_4921c/MAB_4922	MAB_4921c		probable integral membrane protein	4.04	1,2,3
26	2712704	1	MAB_2670c/MAB_2671	MAB_2670c	*hisD*	Histidinol dehydrogenase(AAB)	4.99	1,3
27	4932689	1	upstream of MAB_4820	MAB_4820		Hypothetical protein	4.84	1,3
28	3737457	2	upstream of MAB_3683c	MAB_3683c	*trpS*	Tryptophanyl-tRNA synthetase	4.18	1,3
29	1984334	1	upstream of MAB_1987	MAB_1987		phosphoheptulonate synthase	4.22	1,3
30	3588702	4	MAB_3543c/MAB_3544	MAB_3544		amino-acyl tRNA deacylase	3.77	1,2,3
				MAB_3543c	*sigH*	RNA polymerase sigma-H factor	2.45	1,2,3
31	1632207	1	upstream of MAB_1603	MAB_1603	*valS*	Valyl-tRNA synthetase	3.52	1,3
32	340031	1	MAB_0342c/MAB_0343,	MAB_0343		Aspartate kinase	3.15	1,2,3
33	2357379	1	upstream of MAB_2305c	MAB_2305c		Hypothetical protein	3.13	1,2,3
34	1129660	2	MAB_1118c/MAB_1119	MAB_1118c		Hypothetical protein	2.90	1,2,3
35	3551647	59	upstream of MAB_3509c	MAB_3509c		Hypothetical protein (uORF of whiB7)	2.52	1,2,3
36	3130952	1	upstream of MAB_3084c	MAB_3084c	*dapA*	tetrahydrodipicolinate synthase (AAB)	2.37	1,3
37	3821725	2	upstream of MAB_3763	MAB_3763		cutinase	2.17	1,2,3
38	3012118	1	MAB_2957/MAB_2958	MAB_2958		Putative transport protein	2.06	1,2,3
39	3470985	3	upstream of MAB_3424c	MAB_3424c		2- isopropyl malate synthase	2.04	1,2,3
40	3820662	13	MAB_3762/MAB_3761c	MAB_3762		permease: transporter	1.92	1,2,3
41	3760436	1	upstream of MAB_3705	MAB_3705		Tet family regulator	1.85	1,2,3
42	2689063	1	upstream of MAB_2647c	MAB_2647c	*trpE*	Anthranilate synthase	1.77	1,3
43	1216748	1	upstream of MAB_1201c	MAB_1201c	*greA*	GreA	1.53	1,2,3
44	3252647	2	upstream of MAB_3211c	MAB_3211c		carbonic anhydrase	1.43	1,2,3
45	4204628	1	MAB_4139/ MAB_4138c	MAB_4139		putative transcriptional regulator, ArsR family	1.30	1,2,3
46	3172940	1	upstream of MAB_3131c	MAB_3131c	*infB*	Initiation factor IF-2	1.26	1,2,3
47	2382586	3	MAB_2329c/MAB_2330	MAB_2329c		hypothetical protein, MspA like porin	1.12	1,2,3
48	2089239	1	Lys tRNA/MAB_2089	MAB_2089		Hypotherical protein	1.02	1,2,3
**Intragenic Peaks**								
49	3507096	4	inside MAB_3463	MAB_3464		Hypotherical protein	8.86	1,2,3
50	3077676	1	inside MAB_3023c	MAB_3022?		Hypotherical protein	1.03	1,2,3
51	2191174	1	N-terminal of MAB_2177	MAB_2177		probable ABC transporter, periplasmic	7.47	1,2,3
52	3670771	1	N-terminal of MAB_3621	MAB_3621		Probable taurine dioxygenase	1.77	1,2,3
53	4941059	2	inside MAB_4827	unknown		n/a	n/a	1,3
**Probable RNAs**								
54	3513806	4	upstream of MAB_3472	MAB_3471		Opposite orientation of motif; antisense RNA detected	ncRNA:1.4	1,2,3
							MAB_3471: 1.4	1,2,3
55	736028	9	MAB_0731/ MAB_0732c	MAB_0732		Wrong direction for both ORFs; antisense RNA detected	ncRNA:99	1,2,3
							MAB_0732c: 10.3	1,2,3
56	3252670	1	upstream of MAB_3211c	opposite direction		Wrong orientation for MAB_3211c; antisense to MAB_3212c?		1,2,3

### Identification of a conserved binding motif and determination of direct targets of WhiB7

**Fig 2:**
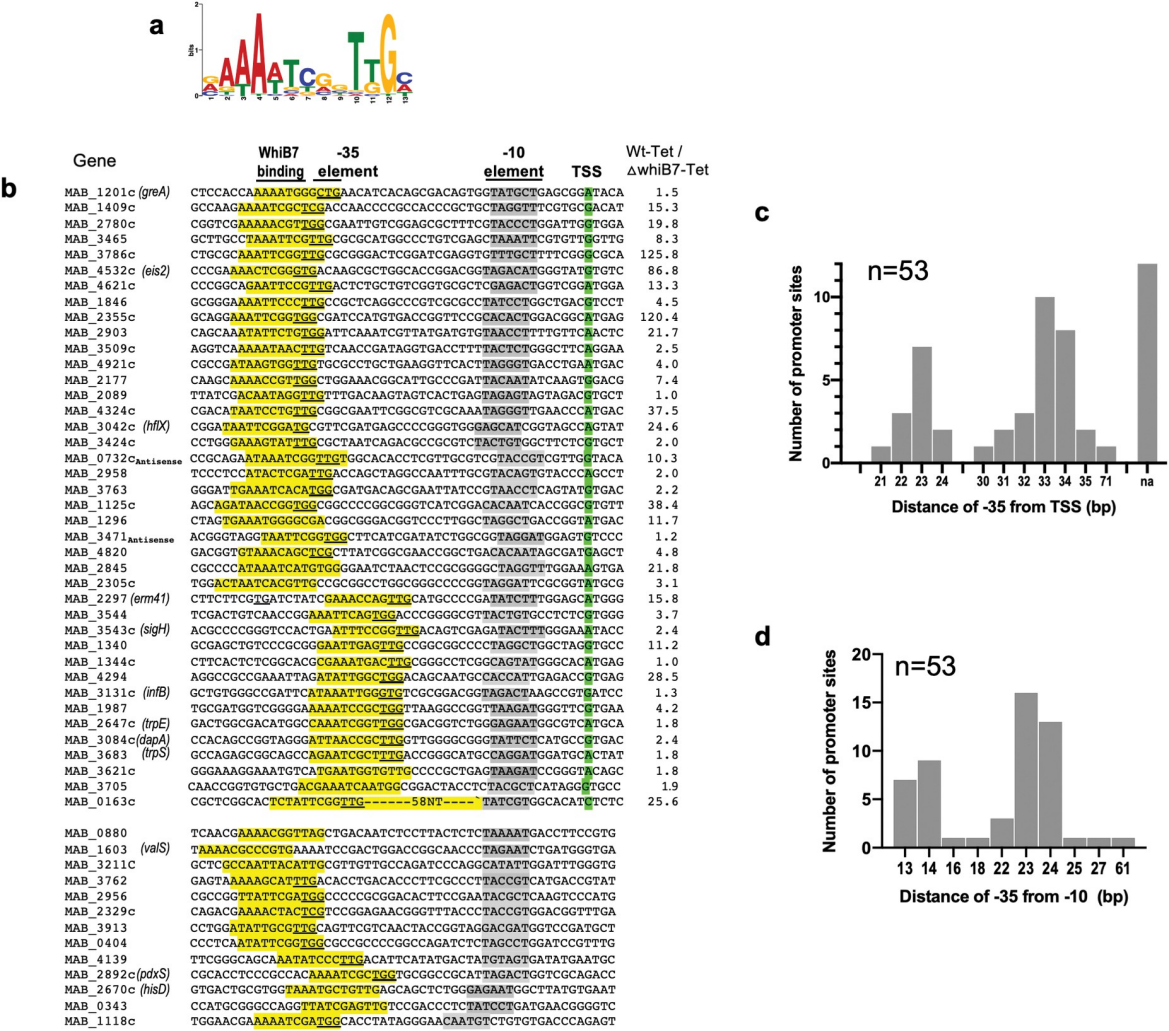
Conserved motif within WhiB7 binding sites. **a)** Sequence logo of enriched motif in WhiB7_FLAG_ bound sites identified using MEME Suite 5.5.1 (MEME E-value = 3.7.0e^-015^) showing the presence of an AT rich sequence separated by 3nts from a -35 site. **b)** Location of WhiB7 motif (yellow), known TSSs (green) and potential -10 sequences (gray) within WhiB7_FLAG_ binding sites. -35 sites are underlined (yellow). Fold downregulation of each genes in *ΔMabwhiB7* compared to wild-type strain is also noted. **c)** Histogram showing the frequency distribution of distances between the -35 sequence and known TSSs. **d)** Histogram showing the frequency distribution of distances between -35 sequence and potential -10 sequences corresponding to the σ^A^/σ^B^ consensus TANNNT.

Next, we analyzed the sequences corresponding to the MabWhiB7 peaks using MEME tools and identified a conserved motif comprised of an AT-rich sequence 3bp upstream of the -35 promoter element in all 56 CHIP-Seq regions (Fig 2A and 2B). The orientation of the conserved WhiB7 binding motif was then used to determine the identity of the genes regulated by WhiB7 binding and are shown in Tables 1 and S2. Using this method, 48 of 51 intergenic WhiB7 peaks were found to potentially regulate 49 *M*. *abscessus genes*. We used the RNAseq dataset to determine if the 49 genes identified above were differentially expressed in the Δ*MabwhiB7* strain when exposed to TET. Table 1 shows that the expression of 40 genes is significantly (*p*_*adj*_ <0.01) downregulated >2-fold in Δ*MabwhiB7*, whereas the expression of 9 genes remains unchanged. The expression of 11 of the direct targets were additionally validated using qPCR (S1 Data). However, none of the genes directly regulated by WhiB7 showed an increase in expression in Δ*MabwhiB7* implying that WhiB7 functions exclusively as a transcriptional activator. We also investigated the 5 intragenic WhiB7 binding peaks closely as they were all associated with a σ^70^ peak. Of these, the WhiB7 binding sites within MAB_2177 and MAB_3621 were located at the beginning of ORFs, suggesting that these genes are likely misannotated. The significance of WhiB7 binding within MAB_3023c, MAB_3463 and MAB_4827 is unknown but could be driving the expression of downstream genes MAB_3022c and MAB_3464. Indeed, we find the expression of MAB_2177 and MAB_3464 to be strongly WhiB7 dependent (Table 1).

Following identification of WhiB7 targets we mapped known transcription start sites (TSSs) with relation to the WhiB7 binding sites [18]. Fig 2B and 2C and S2 Table show that most of the -35 elements were located either 22-23nt or 33–34 nt from the TSS. A sequence corresponding to the -10 consensus sequence of σ^A^/σ^B^ specific promoters (TANNNT) could also be identified either 13–14 nt or 23-24nt downstream of the -35 element and is consistent with previous studies in *M*. *tuberculosis* that demonstrate a cooperative binding between σ^A^ and WhiB7 [15,32] (Fig 2B–2D and Table 2). However, we do not observe a correlation between the degree of dependence of WhiB7 in gene expression and the spacing between -35 and -10 elements.

**Table 2:** Expression of operonic genes regulated by WhiB7.

Gene immediately downstream of WhiB7 binding site	Fold under- represented in △whiB7 mutant	Operonic genes	Gene names	Function	Fold under represented in △*whiB7* mutant
MAB_3786c	125.79	MAB_3785c		Lipoprotein LppF	19.21
MAB_1125c	38.44	MAB_1124c		Probable para-aminobenzoate synthase component I	7.69
MAB_0163	25.62	MAB_0162c		hypothetical protein	3.37
MAB_2780c	19.90	MAB_2779c		Glyceraldehyde-3-phosphate dehydrogenase	2.04
		MAB_2778c	*pgk*	phosphoglycerate kinase	3.33
MAB_2297	15.90	MAB_2298	*coaA*	Dephospho-CoA kinase	4.47
MAB_1340	11.23	MAB_1341		hypothetical protein	8.86
MAB_0880	6.94	MAB_0881		tRNA/rRNA methyltransferase	3.67
MAB_2892c	5.08	MAB_2890c	*pdxT*	Glutamine amidotransferase PdxT	5.59
		MAB_2891c		acyl-CoA thioesterase II	5.29
MAB_1846	4.57	MAB_1847		Metalloprotease	5.44
		MAB_1848		Hypothetical protein	3.63
MAB_3683c	4.19	MAB_3682c		membrane protein	2.13
MAB_1603	3.52	MAB_1604		folylpolyglutamate synthase FolC	11.50
MAB_0343	3.15	MAB_0344	*asd*	50S ribosomal protein L28	4.31
		MAB_0345		Hypothetical protein	2.79
MAB_3084	2.37	MAB_3083	*rnj*	ribonuclease J	3.33
MAB_3763	2.17	MAB_3764		epoxide hydrolase EphA	2.02
MAB_2958	2.05	MAB_2959		Putative N-acetyltransferase, Rim I related	2.03
MAB_2647c	1.76	MAB_2646c		Membrane protein in trp biosynthesis	2.41
MAB_3131c	1.26	MAB_3130	*rbfA*	Ribosome-binding factor A (RbfA)	2.63

We then determined if the genes that are likely to be within the same transcription unit as the direct targets of WhiB7 were differentially expressed in wild-type *M*. *abscessus* when compared to Δ*MabwhiB7* (RNAseq). We identified 21 genes whose expression was WhiB7 dependent and were likely to be upregulated directly by WhiB7 binding upstream of the operon (Table 2). Therefore, binding of WhiB7 to 56 chromosomal locations unequivocally accounted for the WhiB7 dependent upregulation of 72 genes (49 intergenic, 2 intragenic and 21 operonic genes).

### Regulation of non-coding RNAs by MabWhiB7

The WhiB7 motif at 3 intragenic locations were found to be oriented in a direction opposite to that of the ORFs within the region and are suggestive of driving the expression of ncRNAs (Fig 1C and Table 1). From the RNAseq dataset we detected ncRNAs in the region between MAB_0731 and MAB_0732c (ncRNA1) and between MAB_3471 and MAB_3472 (ncRNA2) consistent with a previous report [18]. The expression of ncRNA1 increases upon treatment with TET and is downregulated ~100-fold (*p*_*adj*_ <0.001) in the Δ*MabwhiB7* mutant (Fig 1D and S1 Data). Interestingly, ncRNA1 is complementary along most of its length to the 3’-UTR of MAB_0732c (Fig 1C). Since the expression of MAB_0732c is downregulated ~10-fold in Δ*MabwhiB7* it is likely that ncRNA1 functions by stabilizing MAB_0732c mRNA and merits further investigation. A differential change in expression of ncRNA2 was not observed in the Δ*MabwhiB7* mutant and its function is not immediately obvious.

### Characterization of direct targets of WhiB7 identifies novel effectors of innate drug resistance

**Fig 3:**
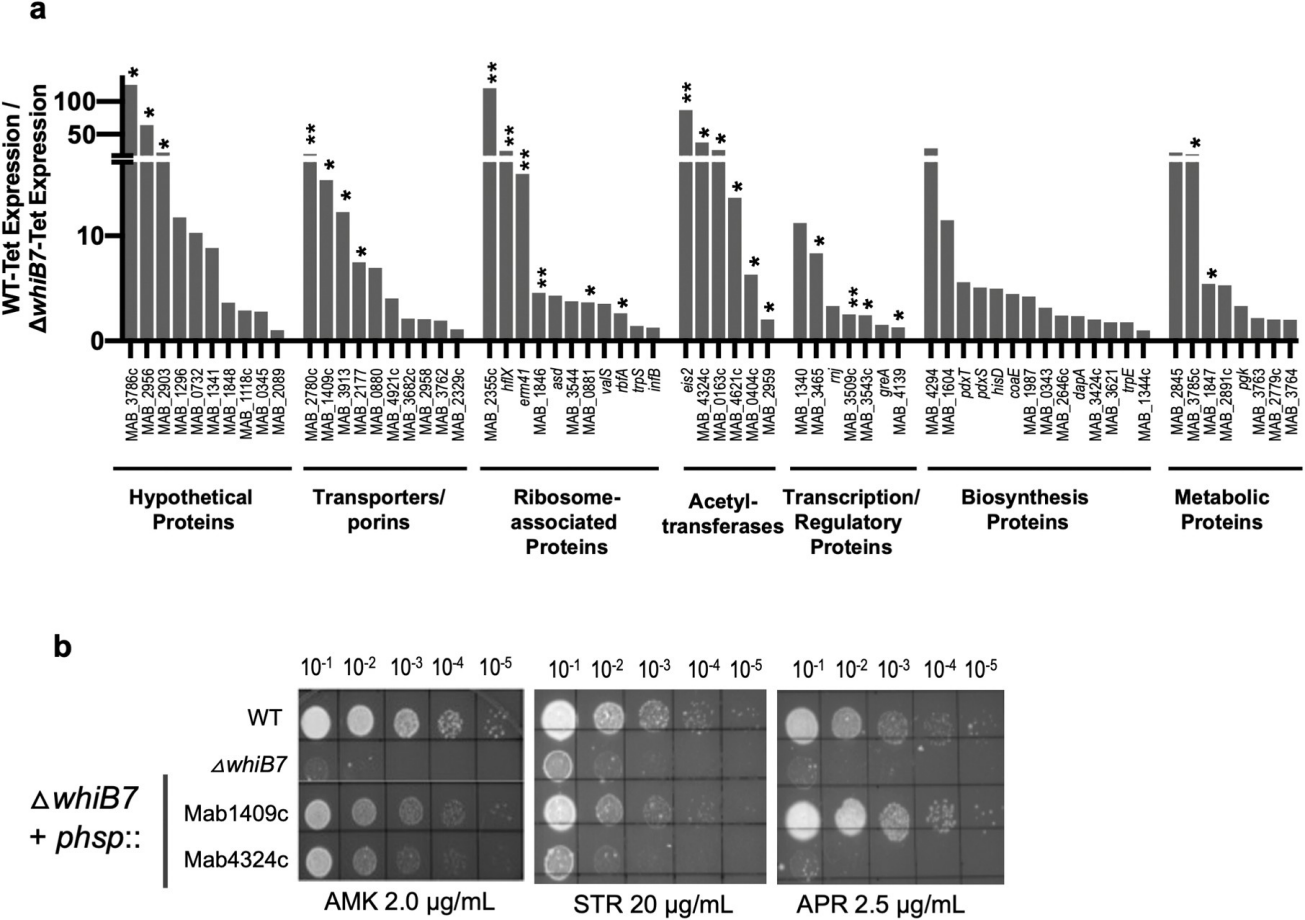
Functional analysis of WhiB7 regulated genes. **a)** Functional classification of genes directly regulated by WhiB7 and the fold down-regulation in a Δ*MabwhiB7* is shown. Genes with known roles in drug resistance is indicated with **. Genes that have been evaluated in this study are indicated with *. **b)** Growth of ten-fold serial dilutions of *M*. *abscessus* ATCC 19977, *ΔMabwhiB7*, and *ΔMabwhiB7* complemented with either MAB_1409c or MAB_4532c on Middlebrook 7H10 OADC containing indicated concentrations of AMK, STR and APR. Data is representative of >3 independent experiments.

The direct targets of WhiB7 belong to a variety of functional categories including genes encoding ribosome associated proteins, transcription regulators, acetyltransferases, transporters, biosynthetic and metabolic enzymes and hypothetical proteins. Of these, 7 genes have been characterized previously and account for the prominent hypersensitivity of Δ*MabwhiB7* to AMK, SPC, macrolide and lincosamide antibiotics (Fig 3A and Table 3); the role of the majority of genes regulated by WhiB7 (either directly or indirectly) is unknown. Here we have exploited the antibiotic hypersensitivity of the Δ*MabwhiB7* strain to evaluate the function of a subset of WhiB7 dependent genes. For this, a gene under investigation was constitutively expressed from a chromosomal location of a Δ*MabwhiB7* strain, followed by analysis of its ability to complement the drug susceptibility of Δ*MabwhiB7*. We have previously used this assay to establish the role of MAB_2780c, *hflX* and MAB_1846 in SPC, macrolide and lincosamide resistance [30]. A spectrum of 8 drugs that target both the 50S and 30S ribosomal subunits- amikacin (AMK), tigecycline (TIG), streptomycin (STR), spectinomycin (SPC), apramycin (APR), erythromycin (ERT), clarithromycin (CLA) and clindamycin (CLIN)- were used to evaluate the function of 15 genes belonging to different categories:

*Acetyltransferases*: Acetyltransferases are known to transfer an acetyl group to diverse substrates from small molecules such as aminoglycosides and mycothiol to macromolecules [33]. Aminoglycosides, classified as 2-DOS (APR, AMK, KAN) and non-DOS antibiotics (STR) based on their chemical structure, target slightly different portions of the 16S rRNA within the 30S ribosomal subunit [34]. Resistance to aminoglycosides is commonly mediated by drug modification by acetyltransferases, phosphotransferases and nucleotidyltransferases [34]. In *M*. *abscessus*, the WhiB7 dependent N-acetyltransferases MAB_4395 (*aac2*) and MAB_4532c (*eis2*), are known to confer resistance to kanamycin B/tobramycin-/dibekacin and AMK respectively [28]. MAB_2385, a phosphotransferase outside the WhiB7 regulon, is the only known determinant of STR resistance [35]. The *M*. *abscessus* genome encodes a large number of additional acetyltransferases and phosphotransferases several of which are included within the WhiB7 regulon and 6 are direct targets. We tested 4 acetyltransferases with unknown functions and a phosphotransferase of which MAB_4324c showed only a modest ability to complement the AMK sensitivity of Δ*MabwhiB7* (Figs 3B and S2A–S2D). Interestingly, the AMK sensitivity of a ΔMAB_4324c deletion strain was previously shown to be indistinguishable from that of WT bacteria; purified Mab4324c nonetheless showed mild acetylation of AMK [36]. These results are consistent with the modest AMK resistance conferred by MAB_4324c in the AMK hypersensitive background of Δ*MabwhiB7* and is suggestive of a relatively minor role of MAB_4324c in AMK resistance of *M*. *abscessus*.

*Transporters*: The *M*. *abscessus* genome encodes a large number of putative transporters and efflux pumps, several of which are within the WhiB7 regulon and are inducible by ribosome targeting antibiotics; 10 were found to be direct targets of WhiB7 (Fig 3A and Table 1). However, the mere induction of a transporter does not confirm its role in drug resistance and the functions of the vast majority of efflux pumps are unknown. We evaluated the function of 3 putative transporters–MAB_1409c, MAB_2177 and MAB_3913. Of these, MAB_1409c, a putative MFS efflux pump, effectively complements APR sensitivity of Δ*MabwhiB7* (Fig 3B). A mild complementation of AMK and STR sensitivity of Δ*MabwhiB7* by MAB_1409c was also observed and is consistent with a previous study (Fig 3B) [11]. Future characterization of MAB_1409c will enable determination of its substrate specificity and mechanisms of efflux.

Complementation of antibiotic sensitivity of Δ*MabwhiB7* by the remaining 10 genes encoding hypothetical proteins, ribosome associated proteins and other enzymes could not be detected (Figs S2A–S2D and 3A and Table 3).

**Table 3:** Summary of gene functions within the direct regulon of WhiB7.

Gene Regulated	Gene description	Role in Drug resistance
MAB_3509c	Hypothetical protein (uORF of whiB7)	Expression of whiB7 (22)
MAB_1846	Putative ABC transporter ATP-binding protein	Lincosamide (L) (24)
MAB_2297	erm41	MLSKO (34)
MAB_2355c	Putative ABC transporter ATP-binding protein	Macrolide (M) (27)
MAB_2780c	MFS transporter	SPC (31)
MAB_3042c	HflX	Macrolide/lincosamide (25)
MAB_3543c	RNA polymerase sigmaH factor	TIG (32)
MAB_4532c	Acetyltransferase	AMK (26)
MAB_1409c	Putative drug antiporter protein precursor (MFS)	**APR, STR, AMK**
MAB_3543c	RNA polymerase sigma-E factor	**AMK, STR, SPC, APR**
MAB_4324c	Putative acetyltransferase, GNAT	**AMK**
MAB_0163c	Aminoglycoside phosphotransferase	Not detected
MAB_0404c	Putative GNAT acetyltransferase	Not detected
MAB_0881	tRNA/rRNA methyltransferase	Not detected
MAB_1847	Metalloprotease	Not detected
MAB_2177	probable ABC transporter, periplasmic	Not detected
MAB_3785c	Lipoprotein LppF	Not detected
MAB_2903	Hypothetical protein	Not detected
MAB_2956	hypothetical protein—GATB Yqey superfamily	Not detected
MAB_2959	Putative N-acetyltransferase, Rim I related	Not detected
MAB_3130	Ribosome-binding factor A (RbfA)	Not detected
MAB_3465	Putative sulfate transporter/antisigma-factor antagonist	Not detected
MAB_3786c	Hypothetical protein	Not detected
MAB_3913	Putative translocator (RhtB family- efflux?)	Not detected
MAB_4139	putative transcriptional regulator, ArsR family	Not detected
MAB_4621c	Putative acetyltransferase	Not detected

### σ^H^ regulates secondary pathways required for intrinsic resistance to TIG, AMK, APR and STR

**Fig 4:**
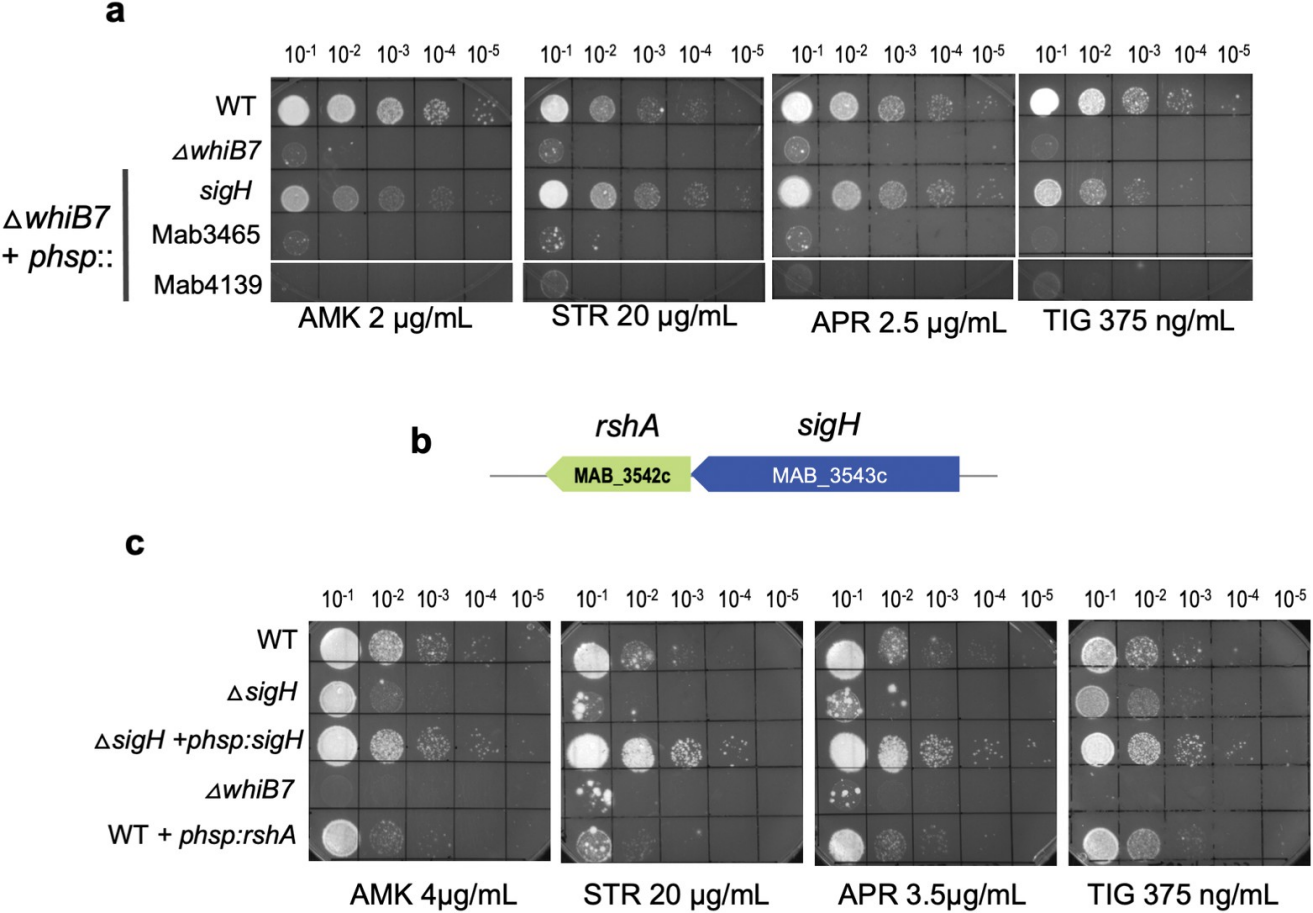
Functional analysis of transcriptional regulators within the direct regulon of WhiB7. **a)** Growth of ten-fold serial dilutions of *M*. *abscessus* ATCC 19977, Δ*MabwhiB7*, and Δ*MabwhiB7* complemented with either MAB_3453c or MAB_3465 on Middlebrook 7H10 OADC containing indicated concentrations of AMK, STR, TIG, SPC and APR. Data is representative of >3 independent experiments. **b)** Genomic organization of *sigH* (MAB_3543c) and its anti-sigma factor *rshA* (MAB_3542c). **c)** Growth of ten-fold serial dilutions of *M*. *abscessus* ATCC 19977, Δ*MabwhiB7*, Δ*MabsigH*, Δ*MabsigH* complemented with *sigH* and wild-type *M*. *abscessus* overexpressing *rshA* on Middlebrook 7H10 OADC containing indicated concentrations of AMK, STR, TIG, and APR. Data is representative of >3 independent experiments.

The CHIP-Seq data set shows that WhiB7 directly affects expression of 72 genes within the WhiB7 regulon of 181 genes. This suggests a hierarchical control of gene expression in which WhiB7 directly affects transcription of 72 genes, while the remaining genes are indirectly WhiB7 dependent and are likely controlled by one or more transcriptional regulators contained within the direct targets of WhiB7. We therefore tested three transcriptional regulators that are direct targets of WhiB7- MAB_3543c (*sigH*), MAB_3465 (a putative anti-sigma factor antagonist) and MAB_4139 (ArsR) for their ability to complement the drug sensitive phenotype of Δ*MabwhiB7*. Expression of *sigH*, but not MAB_4139 or MAB_3465, effectively complemented the STR and APR sensitivity of Δ*MabwhiB7* and mildly complemented the AMK and TIG sensitivity of Δ*MabwhiB7* (Figs 4A and S2); its effect on 50S targeting antibiotics was negligible (S3 Fig).

To study the role of *sigH* in intrinsic antibiotic resistance of *M*. *abscessus*, we constructed an isogenic deletion of *sigH* (MAB_3543c) in *M*. *abscessus* ATCC 19977. The Δ*MabsigH* strain was mildly sensitive to AMK and TIG in comparison to Δ*MabwhiB7*, suggesting a modest contribution of *sigH* to AMK and TIG resistance (Fig 4C). In contrast, Δ*MabsigH* and Δ*MabwhiB7* displayed comparable sensitivity to STR and APR (Fig 4C). The phenotype of Δ*MabsigH* is consistent with the complementation results in Fig 4A. Furthermore, overexpression of the regulator of σ^H^, *rshA*, in wild-type bacteria recapitulated the drug sensitivity of Δ*MabsigH*, a phenotype compatible with increased levels of RshA sequestering σ^H^ leading to antibiotic sensitivity (Fig 4B and 4c). Our results are consistent with previous reports which demonstrate that point mutations in *sigH* and its anti-sigma factor, *rshA*, are associated with decreased TIG sensitivity [37,38] and indicate an involvement of σ^H^ in aminoglycoside resistance.

**Fig 5:**
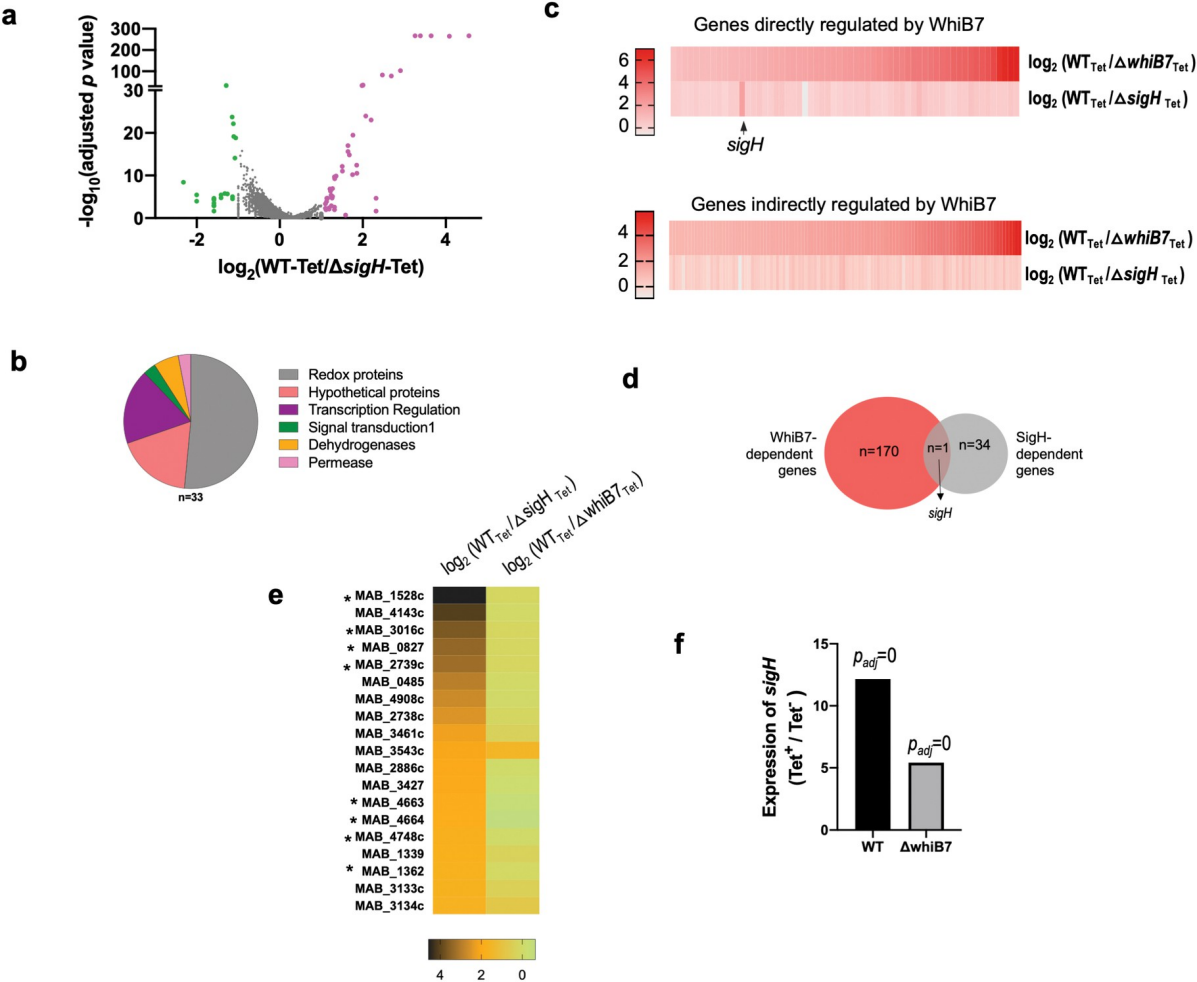
Evaluation of the role of *sigH* in *M*. *abscessus* drug resistance. **a)** Volcano plot of differentially expressed genes in *M*. *abscessus* ATCC19977 wild-type and Δ*MabsigH* strains upon exposure to TET (16μg/mL for 45 mins) determined by RNASeq. Four biological replicates were used of each sample. Genes differentially upregulated >2-fold in wild-type are indicated in purple, and genes downregulated >2-fold in wild-type are indicated in green. **b)** Pie chart of functional categories of σ^H^ regulated genes. **c)** Heat map showing differential expression of WhiB7 regulon genes in Δ*MabsigH* and Δ*MabwhiB7* strains exposed to TET (16μg/mL for 45 mins). **d)** Venn diagram showing overlap of the WhiB7 and σ^H^ regulons. **e)** Heat map showing differential expression of the σ^H^ regulon genes (downregulated >2-fold in Δ*MabsigH*) in Δ*MabsigH and* Δ*MabwhiB7* backgrounds upon exposure to TET (16μg/mL for 45 mins). **f)** Expression of *sigH* in wild-type and Δ*MabwhiB7* strains upon exposure to TET (16μg/mL for 45 mins) using RNAseq.

The above results are suggestive of a scenario in which expression of *sigH* is directly activated by WhiB7, which is then required for expression of a subset of the indirect targets of WhiB7. We therefore determined the global changes in gene expression in a Δ*MabsigH* strain treated with sublethal doses of TET and compared it to a wild-type strain. We identified 33 genes (not including *sigH*) that were differentially regulated > 2-fold (*p*_*adj*_ <0.01) (Fig 5A and 5B and S1 Data) and expected that these would include genes within the WhiB7 regulon. Surprisingly, deletion of *sigH* did not significantly impact (>2-fold; *p*_*adj*_ <0.01) the expression of any of the genes within the WhiB7 regulon implying that the indirect targets of WhiB7 are not dependent on σ^H^ (Fig 5C and 5D and S1 Data). Additional comparison with a previous *M*. *tuberculosis* σ^H^ CHIP-Seq dataset confirmed that besides MAB_3645 and MAB_2850c, none of the indirect targets of WhiB7 could potentially be σ^H^ regulated [39]. Furthermore, the expression of the 33 σ^H^ dependent genes were largely unchanged in a Δ*MabwhiB7* strain (Fig 5E). These results were confounding since overexpression of *sigH* in Δ*MabwhiB7* either partially, or significantly, complemented the drug sensitivity of Δ*MabwhiB7* (Fig 4A). We hypothesized that overexpression of *sigH* in the drug hypersusceptible Δ*MabwhiB7* background can modestly complement the TIG and aminoglycoside sensitivity of Δ*MabwhiB7* and implies that a subset of genes within the σ^H^ regulon functions in the innate resistance to these antibiotics.

### MAB_4664 and MAB_1362 within the σ^H^ regulon influence intrinsic antibiotic susceptibility

**Fig 6:**
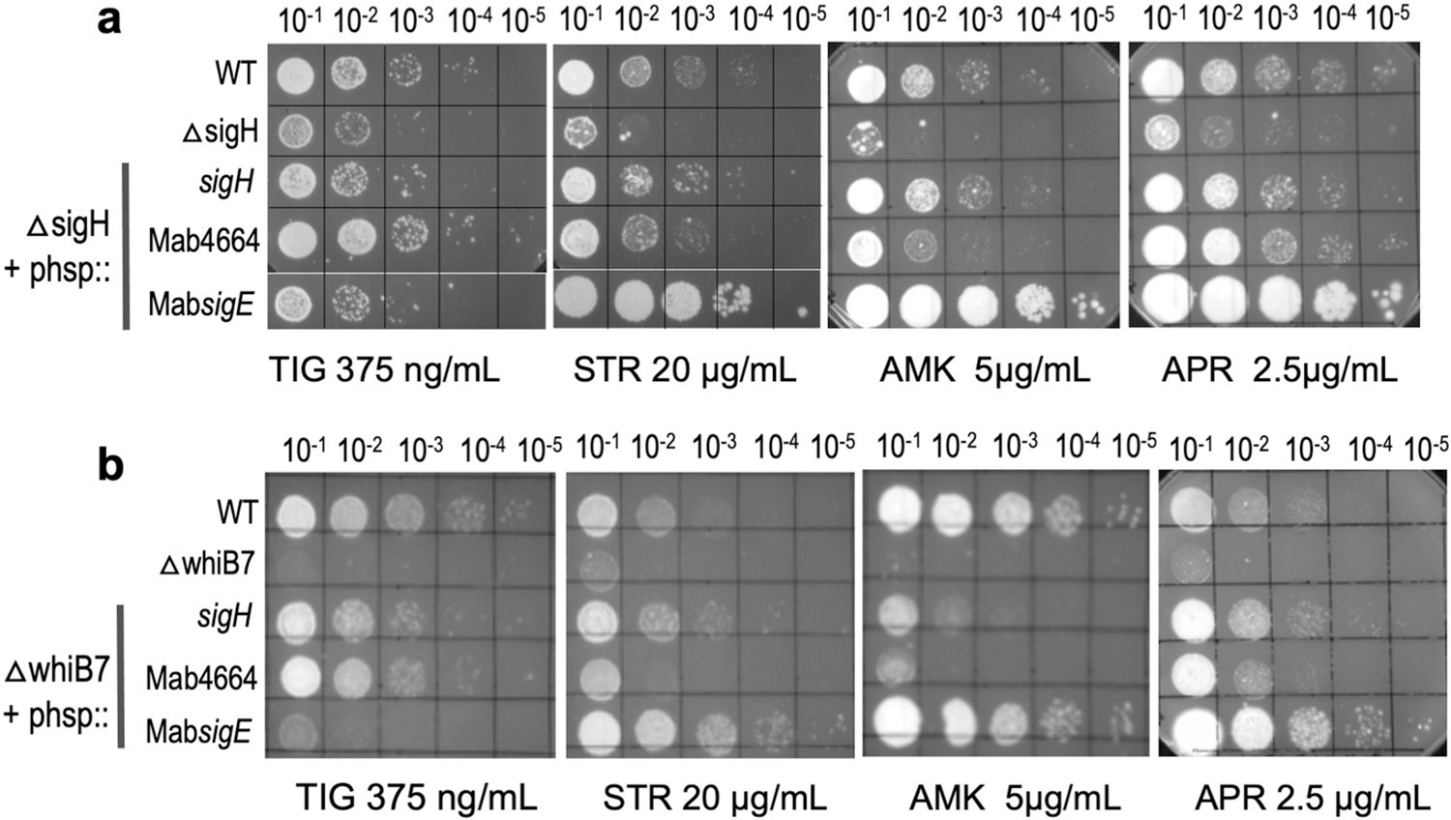
The *sigH* dependent genes, MAB_4664 and MAB_1362, confer drug resistance. **a)** Growth of 10-fold serial dilutions of *M*. *abscessus* ATCC 19977, *ΔMabsigH* and *ΔMabsigH* complemented with *sigH*, *MAB_4664 and MAB_1362 (sigE)* on Middlebrook 7H10 OADC containing indicated concentrations of AMK, STR, TIG, and APR. Data is representative of >3 independent experiments. **b)** Growth of 10-fold serial dilutions of *M*. *abscessus* ATCC 19977, *ΔMabwhiB7* and *ΔMabwhiB7* complemented with *sigH*, *MAB_4664 and MAB_1362 (sigE)* on Middlebrook 7H10 OADC containing indicated concentrations of AMK, STR, TIG, and APR. Data is representative of >3 independent experiments.

Approximately half of the 33 gene Mab σ^H^ regulon is represented by proteins involved in redox pathways; the remaining belong to diverse functional categories (Fig 5B). To determine which of the σ^H^ dependent genes mediate resistance to TIG and aminoglycosides, we evaluated the function of 8 genes encoding proteins belonging to different functional categories–oxidoreductases (MAB_1528c, MAB_ 3016c, MAB_0827, MAB_2739c, MAB_4748c), hypothetical proteins (MAB_4663, MAB_4664) and a regulatory protein (MAB_1362/*sigE*). S4 Fig shows that none of the oxidoreductases tested could complement the drug-sensitive phenotype of Δ*MabsigH*. Overexpression of MAB_4664, a gene encoding a hypothetical protein with no apparent sequence homologue, was found to complement the TIG and aminoglycoside sensitivity of Δ*MabsigH* and Δ*MabwhiB7* to varying extents (Fig 6A and 6B). In addition, overexpression of MAB_*sigE* in Δ*MabsigH* and Δ*MabwhiB7* backgrounds greatly decreased the susceptibility of the mutant strains to STR, AMK and APR but not to TIG. This suggests a hierarchical control of gene expression within the σ^H^ regulon, in which MAB_4664 expression is independent of σ^E^ and is controlled by σ^H^ alone, whereas σ^E^ controls expression of genes within the σ^H^ regulon that determine innate susceptibility to AMK, STR and APR (S5C Fig). A comparison with the previously published Mtbσ^H^ CHIP-Seq data suggests that additionally *sigH*, *sigE*, *sigB*, MAB_2739c, MAB_1527, MAB_1572 are also likely direct targets of σ^H^ [39]. An analysis of the upstream regions of σ^H^ dependent genes using MEME tools displayed the presence of a canonical σ^H^/σ^E^ motif previously described in *M*. *tuberculosis* (S5A and S5B Fig) [39,40]. Curiously we note a variability in the nucleotide immediately downstream of the conserved -35 GGAA motif within the σ^H^ regulated genes; the direct targets of σ^H^ contain a G/C residue whereas the remaining genes with an T/A at that location appear to be regulated by σ^E^ and/or σ^H^ (S5B Fig).

## Discussion

Herein we demonstrate using CHIP-Seq that *M*. *abscessus* WhiB7 binds to 56 chromosomal locations. Using a combination of RNAseq and MEME analysis tools we then determine the identity of genes directly regulated by WhiB7 binding. Whilst the WhiB7 regulon of 181 genes comprises of 11 genes that are downregulated, none of the 72 genes that are direct targets of WhiB7 are downregulated, confirming that WhiB7 functions exclusively as a transcriptional activator. The direct targets of WhiB7 comprise genes with diverse functions, a third of which encode transporters, acetyltransferases and ribosome associated proteins/proteins involved in amino-acid biosynthesis. Of the 72 direct targets, the function of only 7 have been clearly elucidated and account for the observed hypersensitivity of Δ*MabwhiB7* to macrolides, lincosamide, AMK and SPC. The function of the remaining genes within the regulon, as well as the WhiB7 dependent effectors of STR, APR and TIG resistance, remain largely unknown [26–28,30,35,41]. We used a complementation assay to elucidate the role of 18 genes that included 5 putative acetyltransferases, 3 putative transporters, 3 transcription regulators, 3 hypothetical genes, 2 ribosome associated proteins and 2 metabolic enzymes, all of which were significantly drug inducible. Of these, only MAB_1409c, MAB_4324c and MAB_3543c partially complemented the sensitivity of ΔMab*whiB7* to TIG and aminoglycosides. The majority of the WhiB7 targets tested here do not appear to be required for antibiotic resistance phenotypes. This may reflect a limitation in the assay where i) an insufficient number of antibiotics have been tested and ii) multigene interactions were not evaluated. Alternatively, it is possible that only a fraction of genes within the WhiB7 regulon is involved in drug resistance, of which, the prominent players have previously been described and others such as MAB_1409c and MAB_4324c have small contributions to intrinsic resistance. The observed antibiotic hypersensitivity of ΔMab*whiB7* is therefore likely to be an additive effect of these individual contributions. Notably, ΔMabwhiB7 is moderately STR and TIG sensitive; nonetheless no single gene has been identified that can account for this phenotype suggesting that multiple genes with small effects could cumulatively affect resistance to these drugs. Additionally, some genes within the WhiB7 regulon could also be involved in pathways other than antibiotic resistance. Since induction of *whiB7* is brought about by stalled ribosomes on the uORF, it is not inconceivable that any stress that causes ribosome stalling will result in *whiB7* induction. Indeed, induced expression of *whiB7* in *M*. *tuberculosis* has been demonstrated under nutrient starvation, heat shock and low iron conditions [21]. A recent report from the Rock laboratory elegantly demonstrates a requirement of WhiB7 in response to alanine starvation [42]. The inclusion of amino acid biosynthetic genes, tRNA synthetases as well as acetyltransferases within the regulon further support the involvement of the WhiB7 regulon in multiple stress responses [43].

WhiB7 directly regulates the expression of about half of the genes within its regulon which suggests that a transcriptional regulator directly induced by WhiB7 can subsequently activate the next tier of genes within the regulon. Surprisingly, there were few transcriptional regulators that were directly regulated by WhiB7. MAB_3465, an anti-anti sigma factor, although a potentially interesting candidate, neither complemented the drug sensitivity of Δ*MabwhiB7*, nor resulted in a global change in gene expression when expressed in wild-type bacteria (S2 Data). The ability of σ^H^ to complement TIG and aminoglycoside sensitivity of Δ*MabwhiB7* suggested that a subset of genes within the WhiB7 regulon were under the transcriptional control of σ^H^. Contrary to this expectation, RNAseq data demonstrated that the expression of the indirect targets of WhiB7 was σ^H^ independent. It therefore remains to be determined how the expression of ~50% of the WhiB7 regulon is induced in *M*. *abscessus*. While it is possible that some WhiB7 binding peaks have escaped detection in our experiments, it is an unlikely explanation, since we have been unable to detect additional peaks in CHIP-Seq experiments utilizing two different locations of the FLAG tag as well as constitutive and drug-inducible WhiB7 expression. Curiously we observe a striking absence of σ^A^ binding sites upstream of most of these indirect targets of WhiB7 which may suggest the involvement of an alternate sigma-factor that is post-transcriptionally activated in a WhiB7 dependent manner. Additionally, the indirect targets of WhiB7 could include genes that are induced as an adaptive response of bacteria to increased stress in the absence of WhiB7-dependent resistance determinants.

If σ^H^ is required for transcription of genes outside the WhiB7 regulon, why then did overexpression of σ^H^ complement the drug sensitivity of Δ*MabwhiB7*? We note that σ^H^ modestly complemented AMK and TIG sensitivity of Δ*MabwhiB7*; moreover, a *ΔMabsigH* strain was only mildly AMK and TIG sensitive compared to Δ*MabwhiB7* suggesting that σ^H^ plays a minor role in the intrinsic resistance to these drugs. In contrast, *ΔMabsigH* and *ΔMabwhiB7* were equally susceptible to STR and APR suggesting that resistance determinants to these antibiotics are distributed in both regulons. Our results imply that some genes within the σ^H^ regulon confer low levels of resistance to aminoglycosides and TIG, and that the effects of these genes become pronounced when overexpressed in a hypersusceptible Δ*MabwhiB7* background. One such gene is MAB_4664 whose mechanism of action is unknown; the presence of a predicted transmembrane domain and involvement in resistance to multiple antibiotics is suggestive of a role in efflux. In addition, a large majority of the σ^H^ regulated genes are involved in redox- related pathways consistent with a previous definition of the regulon in *M*. *abscessus* using a mutant *rshA* strain as well as in *M*. *tuberculosis* [39,44]. Coincidentally, several antibiotics, including aminoglycosides, have been shown to induce redox-related changes as a secondary aspect of their lethality [45]. It is conceivable that redox proteins encoded within the σ^H^ regulon can mitigate this ancillary effect of antibiotics; consequently, the influence of σ^H^ on antibiotic resistance is also modest.

**Fig 7:**
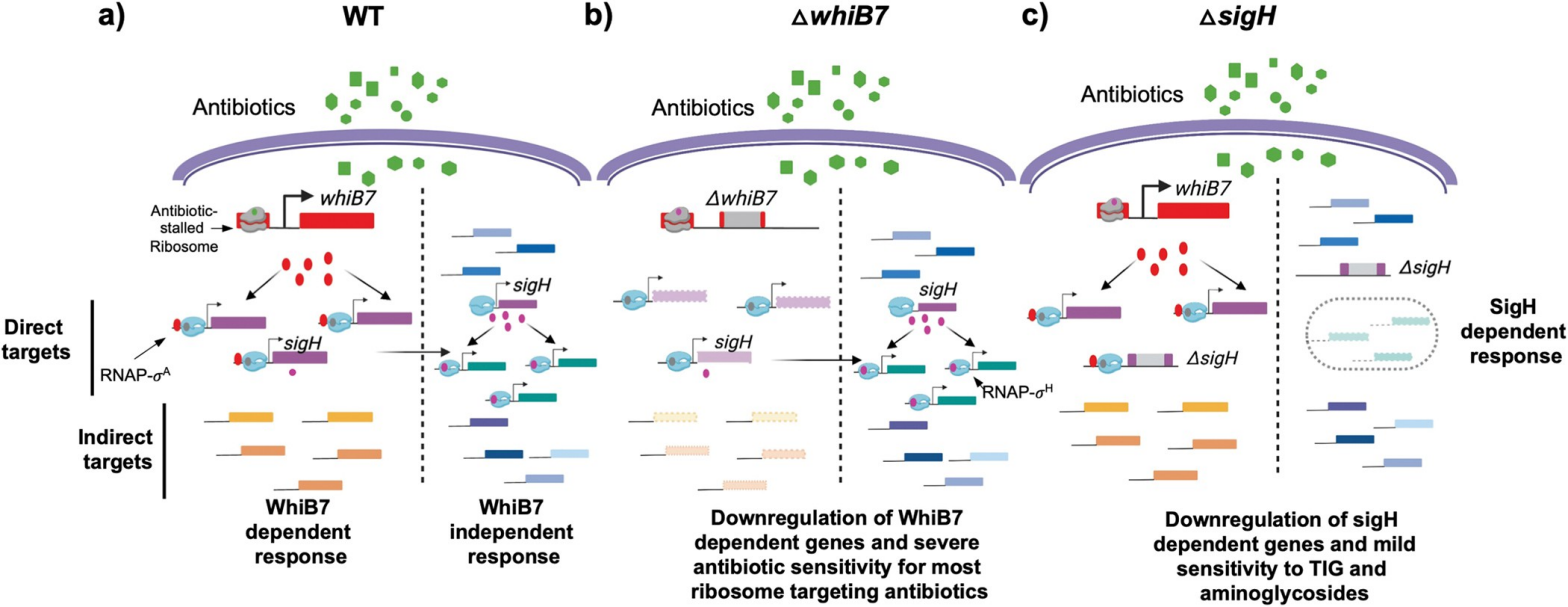
Model showing a cross-talk between WhiB7 and σ^H^ regulons. **(a) Global reprogramming of gene expression in wild type bacteria** by ribosome targeting antibiotics (green) occurs via WhiB7 dependent and independent pathways. WhiB7 regulated genes are shown in purple (direct targets) and orange (indirect targets). Genes induced independent of WhiB7 are shown in green and blue. Induction of *sigH* is antibiotic inducible and WhiB7 independent but is augmented 2-fold in the presence of WhiB7. (**b) Pattern of gene induction in a Δ*MabwhiB7* background.** Downregulated genes are shown in faded purple and orange shades. Induction of *sigH* and its regulon proceeds in ΔMabwhiB7 despite the absence of WhiB7 activation. **(c) Pattern of gene induction in a Δ*MabsigH* background**. Deletion of *sigH* results in downregulation of the *sigH* regulon. The WhiB7 regulon remains unchanged.

The activation of *sigH* expression by WhiB7 appears to be conserved in actinomycetes as the *Streptomyces coelicolor sigR* (the *sigH* homologue) is similarly under WhiB7_Sc_ control [46]. However, the spectrum of resistance conferred by σ^H^ and σ^R^ in the two genera is different and implies the induction of distinct genes. Furthermore, CHIP-Seq analysis of WhiB7 in Streptomyces revealed the presence of 830 binding sites regulating at least 312 genes, which is vastly different from that observed in *M*. *abscessus* despite the presence of significant overlaps [47]. While these could be attributed to differences in methods used to immunoprecipitate WhiB7, it is also possible that the WhiB7 regulon is indeed different in these bacteria. A closer inspection also revealed that expression of Mab*sigH* is ~12-fold induced by TET in wild-type bacteria but is also induced ~ 5-fold in Δ*MabwhiB7*; the transcription of *sigH* is therefore antibiotic inducible even in the absence of WhiB7. (Fig 5F). Since the σ^H^ regulon is unchanged in the Δ*MabwhiB7* strain, it suggests that the levels of functional σ^H^, which is additionally controlled at the post-translational level by anti-σ^H^, does not change significantly in the Δ*MabwhiB7* strain, despite a 2-fold reduction of *sigH* transcript. (Fig 7). Further work is needed to explore if WhiB7 activated expression of *sigH* results in isoforms of σ^H^ with varying stability as seen previously in *S*. *coelicolor* and *M*. *tuberculosis* [46].

## Materials and methods

### Media and bacterial strains

*M*. *abscessus* ATCC19977 strains were grown at 37°C with shaking at 220 rpm in Middlebrook 7H9 (DIFCO) supplemented with 0.05% Tween 80 and 10% OADC. Antibiotics were added to indicated concentrations where required. An isogenic deletion in *MAB_3543c (sigH)* was constructed using recombineering, followed by removal of the zeocin cassette by Cre-mediated recombination at *loxP* sites as described previously [12]. Unmarked deletion mutants were reconfirmed using PCR followed by sequencing of the PCR product. Complementing strains were created by cloning a gene under investigation in pMH94 under the control of the hsp60 promoter followed by integration at the phage L5 *attB* site of an appropriate deletion strain. Mab_whiB7 with a FLAG tag at either the N- or C-terminal was cloned in pMH94 under the control of the hsp60 promoter. Mab_whiB7 with a FLAG tag at the C-terminal was also cloned in pMH94 with its native promoter and upstream regulatory elements contained within a 650 nt leader sequence. All bacterial strains are listed in S1 Table.

### Antibiotic sensitivity assays

Wild type, mutant and complementing strains of *M*. *abscessus* were inoculated at an OD of 0.001 and grown to an OD of 0.7. Cells were tested for drug susceptibility by spotting a 10- fold serial dilution on Middlebrook 7H10 (DIFCO) plates containing the indicated concentration of antibiotics. Spotting on a range of drug concentrations enable determination of the effect of each gene under study.

### RNA preparation and RNA-Seq analysis

Wild type *M*. *abscessus ATCC 19977*, *ΔMabwhiB7*, *ΔMabsigH* were grown to exponential phase (OD = 0.6–0.7) in Middlebrook 7H9-OADC and exposed to 16μg/mL of tetracycline (TET) for 45 minutes. Total RNA was prepared using the Qiagen RNA preparation kit followed by DNAse I treatment. Unexposed samples were used as controls. Approximately 5 μg total RNA samples were treated with the Ribo-Zero rRNA removal procedure (Illumina) to enrich for mRNA. Approximately 500 ng of RNA was used for library preparation using the NextSeq Ultra II (NEB) RNA kit and high throughput sequencing on the Illumina NextSeq platform. The sequence data was analyzed using the reference-based analysis and default parameters on Rockhopper v2.03 in which the data is normalized by upper quartile normalization and transcript abundance is reported as RPKM. Differential gene expression is tested for each transcript and q-values are then reported that control the false discovery rate [48,49]. RNAseq experiments were performed >3 independent times.

### Chromatin immunoprecipitation sequencing (CHIP-Seq) and data analysis

ChIP-Seq was performed as previously described with minor modifications [50]. The Δ*whiB7*::*p*_*nat*_*whiB7*_***C-FLAG***_, Δ*whiB7*::*p*_*hsp*_*whiB7*_***C-FLAG***_ and Δ*whiB7*::*p*_*hsp*_*whiB7*_***N-FLAG***_ strains were grown at 37°C in Middlebrook 7H9 broth with 0.05% Tween 80 and 10% OADC to an OD_600_ = 0.6. The Δ*whiB7*::*p*_*nat*_*whiB7*_***C-FLAG***_ strain was additionally induced with 16μg/mL of tetracycline (TET) for 2hs. This was followed by cross-linking using 1% formaldehyde for 30 min with constant agitation and quenching with 250 mM glycine. The cells were pelleted, washed with TBS buffer and resuspended in buffer 1 (20mM HEPES (pH 7.5), 50mM KCl, 0.5mM DTT, 10% glycerol) containing a protease inhibitor cocktail (Sigma). Cells were harvested and lysed using the CryoMill (Retsch) in buffer 1 followed by sonication for 30 mins at the high setting of the Bioruptor sonicator (Diagenoge). The DNA protein complex was immunoprecipitated with either anti-FLAG monoclonal antibody M2 (Sigma) or anti-σ^70^ antibody (BioLegend) for 18 hours at 4°C and processed as previously described [50]. Each CHIP-Seq experiment was performed using two biological replicates. Genomic DNA libraries enriched for WhiB7 binding were prepared using the NEB NextUltra II Library Prep kit for Illumina followed by sequencing on the Illumina platform (Wadsworth Center, sequencing core facility). Reads were aligned to the reference genome using the Bowtie2 and SamTools algorithms [51]. Regions of enrichment were identified using a custom Python script as described previously [31,32]. Relative enrichment are reported as fold over threshold (FAT) score. The enriched regions were analyzed using MEME Suite 5.5.1 using the default parameters [52].

## Supporting information

S1 Table List of strains used in the study. (PDF)

S2 Table Coordinates of peaks recognized by anti-σ^70^ antibodies (corresponding to σ^A^ and σ^B^ binding), location of TSS sites when available and spacing between -35 of WhiB7 MEME and TSS/-10. (PDF)

S1 Data Differential gene expression in Wt, *ΔMabwhiB7 and ΔMabsigH* in *M*. *abscessus* ATCC 19977 upon exposure to a sublethal dose of TET (16μg/ml for 45 mins).
(XLSX)

S2 Data Differential gene expression in Wt, *and ΔMabwhiB7*::*pMHMab3465*. All datasets are deposited in GEO Accession # GSE233690.
(XLSX)

S1 Fig Ability of WhiB7 tagged strains to complement antibiotic sensitivity of *ΔMabwhiB7*. Growth of ten-fold serial dilutions of *M*. *abscessus* ATCC 19977, *ΔMabwhiB7*, and *ΔMabwhiB7* complemented with either untagged *whiB7*, *whiB7FLAG*_*C-term*_, or *whiB7FLAG*_*N-term*_ expressed from a constitutive promoter or *whiB7FLAG*_*C-term*_ expressed from a native promoter on Middlebrook 7H10 plates containing ERT (7μg/mL). Data is representative of >3 independent experiments.
(PDF)

S2 Fig **(a-d)** Growth of ten-fold serial dilutions of *M*. *abscessus* ATCC 19977, *ΔMabwhiB7*, and *ΔMabwhiB7* complemented with indicated genes on Middlebrook 7H10 plates containing indicated concentrations of antibiotics. Data is representative of >3 independent experiments.
(PDF)

S3 Fig Growth of ten-fold serial dilutions of *M*. *abscessus* ATCC 19977, *ΔMabwhiB7*, and *ΔMabwhiB7* complemented with either MAB_3543c, MAB_3465 and empty vector control on Middlebrook 7H10 plates containing indicated concentrations of 50S targeting antibiotics. Data is representative of >3 independent experiments.
(PDF)

S4 Fig Growth of ten-fold serial dilutions of *M*. *abscessus* ATCC 19977, *ΔMabsigH*, and *ΔMabsigH* complemented with either MAB_3543c, MAB_0827, MAB_1528c, MAB_3016c, MAB_4663, MAB_4748c or MAB_2739c on Middlebrook 7H10 plates containing indicated concentrations of AMK, TIG and STR. Data is representative of >3 independent experiments.
(PDF)

S5 Fig **a)** Sequence logo of enriched motif in σ^H^- dependent genes identified using RNAseq (downregulated >3-fold; p*adj* < 0.01) using MEME Suite 5.5.1. **b)** Location of conserved motif in upstream regions of σ^H^- dependent genes. Fold downregulation of each genes in *ΔsigH* strain is also noted. Difference in base composition of nucleotide immediately downstream to conserved GGAA motif is indicated in green (G/C) and yellow (A/T). **c)** Possible hierarchy within the σ^H^ regulon.
(PDF)
