# Supplementary material for: Hierarchy and interconnected networks in the WhiB7 mediated transcriptional response to antibiotic stress in Mycobacterium abscessus
Source: PLoS Genet. 2023 Dec 6;19(12):e1011060. doi: 10.1371/journal.pgen.1011060 (PMC10727445; doi:10.1371/journal.pgen.1011060)
Supplement: S1 Table — (PDF) [file pgen.1011060.s001.pdf]

Table S1. List of strains used in the study

WT *M. abscessus*	ATCC 19977	ATCC
ΔMabwhiB7	Unmarked deletion mutant of MAB_3508c	#1
ΔMabsigH	Unmarked deletion mutant of MAB_3454c	This study
ΔMabwhiB7::phspwhiB7	*ΔMabwhiB7* containing a chromosomally integrated copy of MAB_3508c (WT) controlled by the hsp60 constitutive promoter	#1
ΔMabwhiB7::phspwhiB7$_{FLAGCterm}$	*ΔMabwhiB7* containing a chromosomally integrated copy of MAB_3508c-FLAG-C-term controlled by the hsp60 constitutive promoter	This study
ΔMabwhiB7::phspwhiB7$_{FLAGCNerm}$	*ΔMabwhiB7* containing a chromosomally integrated copy of MAB_3508c-FLAG-N-term controlled by the hsp60 constitutive promoter	This study
ΔMabwhiB7::p$_{nat}$*whiB7*$_{FLAGCterm}$	*ΔMabwhiB7* containing a chromosomally integrated copy of MAB_3508c-FLAG-C-term controlled by the native *whiB7* promoter	This study
ΔMabwhiB7::phspMab1409c	*ΔMabwhiB7* containing a chromosomally integrated copy of MAB_1409c controlled by the hsp60 constitutive promoter	This study
ΔMabwhiB7::phspMab4324c	*ΔMabwhiB7* containing a chromosomally integrated copy of MAB_4324c controlled by the hsp60 constitutive promoter	This study
ΔMabwhiB7::phspMab3543c	*ΔMabwhiB7* containing a chromosomally integrated copy of MAB_3543c controlled by the hsp60 constitutive promoter	This study
ΔMabwhiB7::phspMab3465	*ΔMabwhiB7* containing a chromosomally integrated copy of MAB_3465 controlled by the hsp60 constitutive promoter	This study
ΔMabwhiB7::phspMab0404c	*ΔMabwhiB7* containing a chromosomally integrated copy of MAB_0404c controlled by the hsp60 constitutive promoter	This study
ΔMabwhiB7::phspMab4621c	*ΔMabwhiB7* containing a chromosomally integrated copy of MAB_4621c controlled by the hsp60 constitutive promoter	This study
ΔMabwhiB7::phspMab2177	*ΔMabwhiB7* containing a chromosomally integrated copy of MAB_2177 controlled by the hsp60 constitutive promoter	This study
ΔMabwhiB7::phspMab2903	*ΔMabwhiB7* containing a chromosomally integrated copy of MAB_2903 controlled by the hsp60 constitutive promoter	This study
ΔMabwhiB7::phspMab2956	*ΔMabwhiB7* containing a chromosomally integrated copy of MAB_3786c controlled by the hsp60 constitutive promoter	This study
ΔMabwhiB7::phspMab3913	*ΔMabwhiB7* containing a chromosomally integrated copy of MAB_3913 controlled by the hsp60 constitutive promoter	This study
ΔMabwhiB7::phspMab4139	*ΔMabwhiB7* containing a chromosomally integrated copy of MAB_4139 controlled by the hsp60 constitutive promoter	This study
ΔMabwhiB7::phspMab4139	*ΔMabwhiB7* containing a chromosomally integrated copy of MAB_4139 controlled by the hsp60 constitutive promoter	This study
ΔMabsigH::phspMab1528c	*ΔMabsigH* containing a chromosomally integrated copy of MAB_1528c controlled by the hsp60 constitutive promoter	This study
ΔMabsigH::phspMab3016c	*ΔMabsigH* containing a chromosomally integrated copy of MAB_3016c controlled by the hsp60 constitutive promoter	This study
ΔMabsigH::phspMab4663	*ΔMabsigH* containing a chromosomally integrated copy of MAB_4663 controlled by the hsp60 constitutive promoter	This study
ΔMabsigH::phspMab4664	*ΔMabsigH* containing a chromosomally integrated copy of MAB_4664 controlled by the hsp60 constitutive promoter	This study
ΔMabsigH::phspMab4748c	*ΔMabsigH* containing a chromosomally integrated copy of MAB_4748c controlled by the hsp60 constitutive promoter	This study
ΔMabsigH::phspMab2739c	*ΔMabsigH* containing a chromosomally integrated copy of MAB_2739c controlled by the hsp60 constitutive promoter	This study
ΔMabsigH::phspMab1362	*ΔMabsigH* containing a chromosomally integrated copy of MAB_1362 controlled by the hsp60 constitutive promoter	This study
WT+ phspMabrshA	*WT M. abscessus* containing a chromosomally integrated copy of MAB_1362 controlled by the hsp60 constitutive promoter	This study

1. Hurst-Hess K, Rudra P, Ghosh P. Mycobacterium abscessus WhiB7 Regulates a Species-Specific Repertoire of Genes To Confer Extreme Antibiotic Resistance. Antimicrob Agents Chemother. 2017;61(11). Epub 2017/09/07. doi: 10.1128/AAC.01347-17. PubMed PMID: 28874378; PubMed Central PMCID: PMCPMC5655061.
