# Supplementary material for: Hierarchy and interconnected networks in the WhiB7 mediated transcriptional response to antibiotic stress in Mycobacterium abscessus
Source: PLoS Genet. 2023 Dec 6;19(12):e1011060. doi: 10.1371/journal.pgen.1011060 (PMC10727445; doi:10.1371/journal.pgen.1011060)
Supplement: S2 Table — (PDF) [file pgen.1011060.s002.pdf]

Table S2: Location of σ^{70} binding sites and TSS relative to WhiB7 binding sites

SigA CHIP-seq peak Coordinates	WhiB7 CHIPseq peak Coordinates	Gene Regulated	TSS	Spacing between motif and TSS	Spacing between motif and -10
3842419	3842377	MAB_3786c	3842397	33	24
2411983	2411990	MAB_2355c	2411993	34	24
4615553	4615555	MAB_4532c	4615566	34	24
3011065	3011060	MAB_2956	na	na	23
4404754	4404776	MAB_4324c	4404783	34	24
3095971	3095974	MAB_3042c	3095977	35	24
2954778	2954769	MAB_2903	2954778	33	23
2828533	2828542	MAB_2780c	2828544	33	24
2345951	2345935	MAB_2297	2345927	22	13
1413694	1413707	MAB_1409c	1413705	33	24
4707208	4707211	MAB_4621c	4707223	32	23
3966094	3966090	MAB_3913	na	na	24
1341898	1341921	MAB_1340	1341922	24	14
3507836	3507827	MAB_3465	3507835	32	23
874942	874984	MAB_0880	na	na	22
405478	405480	MAB_0404c	na	na	23
2944981	2944991	MAB_2892c	na	na	14
1844720	1844715	MAB_1846	1844710	33	23
5030047	5030041	MAB_4921c	5030054	34	24
3588682	3588702	MAB_3544	3588768	25	14
		MAB_3543c	3588704	21	13
1129635	1129660	MAB_1118c	1129677	na	16
3551617	3551647	MAB_3509c	3551635	33	23
3470974	3470985	MAB_3424c	3470992	34	23
3820679	3820662	MAB_3762	3820266	na	24
1216740	1216748	MAB_1201c	1216746	31	22
4204594	4204628	MAB_4139	na	na	18
2383570	2382586	MAB_2329c	na	na	23
2089231	2089239	MAB_2089	2089297	33	23
3012165	3012118	MAB_2958	3012152	32	23
340040	340031	MAB_0343	na	na	13
3172950	3172940	MAB_3131c	3172964	23	13
4370260	4370213	MAB_4294	4370226	23	13
158510	158505	MAB_0163c	158473	71	61
1299265	1299192	MAB_1296	1299233	33	23
1346465	1346473	MAB_1334c	1346466	22	13
1632240	1632207	MAB_1603	na	na	27
1984375	1984334	MAB_1987	1984351	23	14
2689050	2689063	MAB_2647c	2689049	23	14
2712715	2712704	MAB_2670c	na	na	14
3130970	3130952	MAB_3084c	3130981	23	14
3737460	3737457	MAB_3683	3737464	23	14
3670772	3670771	MAB_3621c	3670784	22	13
2191192	2191174	MAB_2177	2191189	34	24
2357392	2357379	MAB_2305c	2357398	34	24
2893879	2893879	MAB_2845	2893900	33	23
4932661	4932689	MAB_4820	4932688	33	23
1136824	1136821	MAB_1125c	1136840	35	25
3821739	3821725	MAB_3763	3821719	34	24
3252664	3252647	MAB_3211c	na	na	23
3760426	3760436	MAB_3705	3760427	24	14
**Probable RNAs**					
3513782	3513806	upstream of MAB_3472	3513798	30	22
736042	736028	MAB_0731/ MAB_0732c	736030	30	22
3252670	3252670	upstream of MAB_3211c	n/a		
