# Supplementary figures and images for: Hierarchy and interconnected networks in the WhiB7 mediated transcriptional response to antibiotic stress in Mycobacterium abscessus
Source: PLoS Genet. 2023 Dec 6;19(12):e1011060. doi: 10.1371/journal.pgen.1011060 (PMC10727445; doi:10.1371/journal.pgen.1011060)

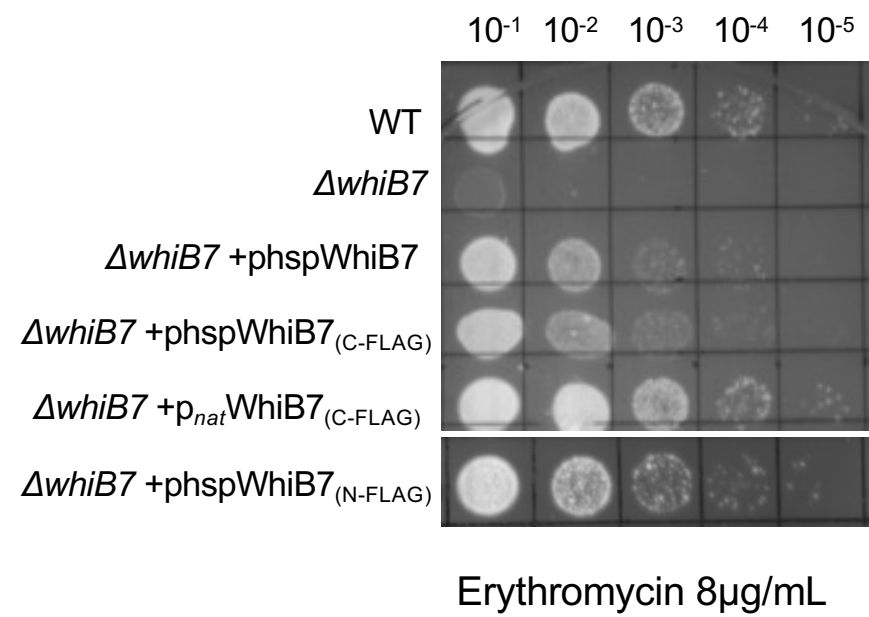

Figure S1

Supplement: S1 Fig — Growth of ten-fold serial dilutions of M. abscessus ATCC 19977, ΔMabwhiB7, and ΔMabwhiB7 complemented with either untagged whiB7, whiB7FLAGC-term, or whiB7FLAGN-term expressed from a constitutive promoter or whiB7FLAGC-term expressed from a native promoter on Middlebrook 7H10 plates containing ERT (7μg/mL). Data is representative of >3 independent experiments. (PDF) [file pgen.1011060.s005.pdf]

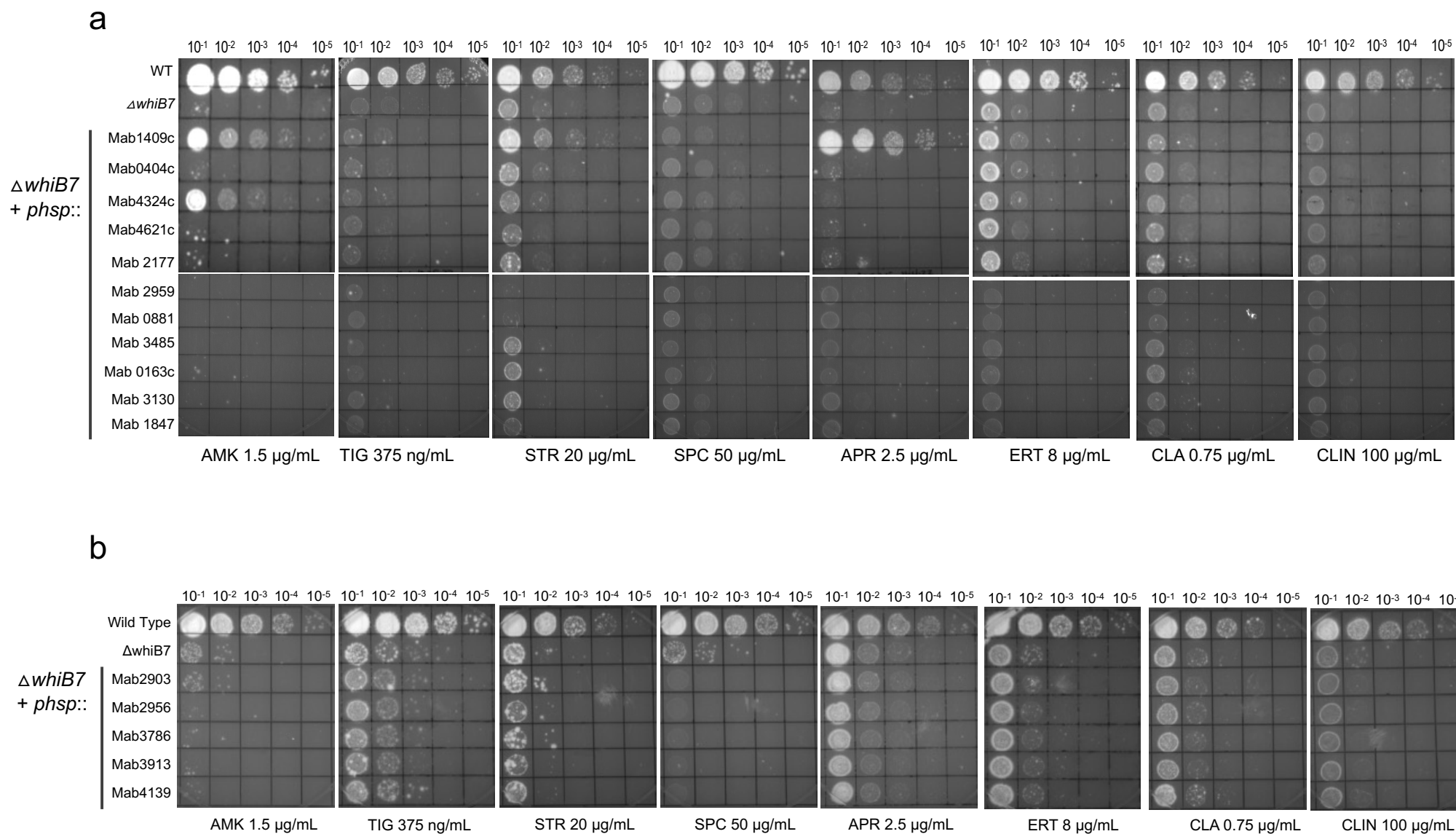

Figure S2

Supplement: S2 Fig — (a-d) Growth of ten-fold serial dilutions of M. abscessus ATCC 19977, ΔMabwhiB7, and ΔMabwhiB7 complemented with indicated genes on Middlebrook 7H10 plates containing indicated concentrations of antibiotics. Data is representative of >3 independent experiments. (PDF) [file pgen.1011060.s006.pdf]

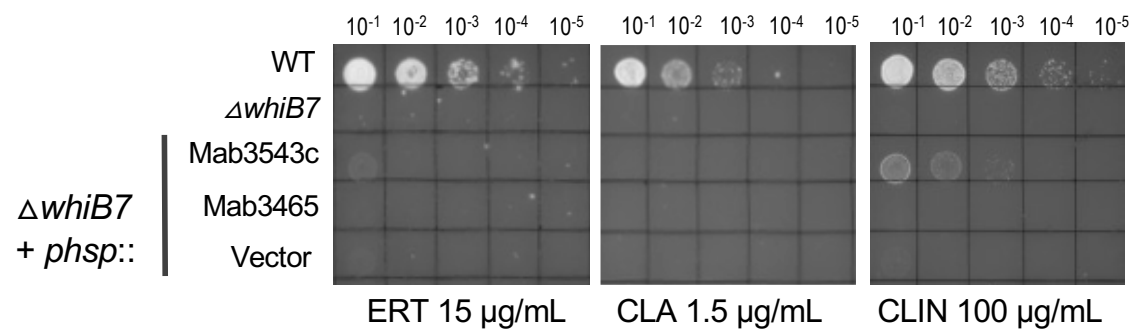

Figure S3

Supplement: S3 Fig — abscessus ATCC 19977, ΔMabwhiB7, and ΔMabwhiB7 complemented with either MAB_3543c, MAB_3465 and empty vector control on Middlebrook 7H10 plates containing indicated concentrations of 50S targeting antibiotics. Data is representative of >3 independent experiments. (PDF) [file pgen.1011060.s007.pdf]

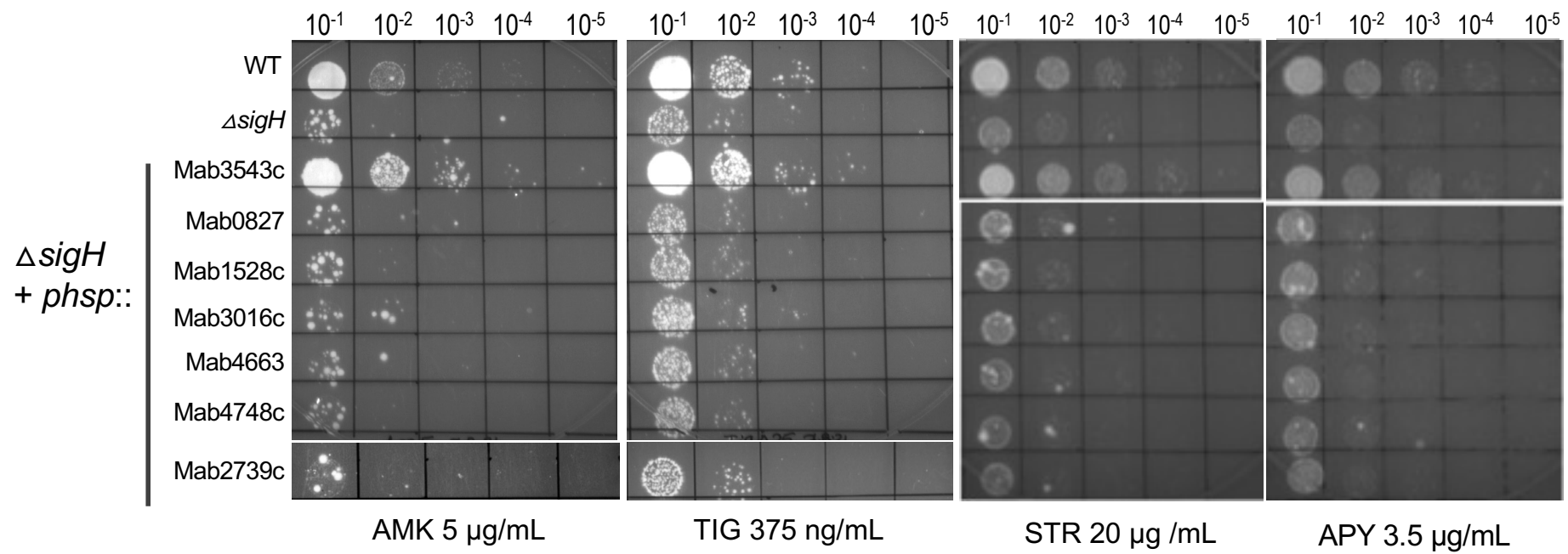

Figure S4

Supplement: S4 Fig — Data is representative of >3 independent experiments. (PDF) [file pgen.1011060.s008.pdf]
