# Supplementary material for: Hierarchy and interconnected networks in the WhiB7 mediated transcriptional response to antibiotic stress in Mycobacterium abscessus
Source: PLoS Genet. 2023 Dec 6;19(12):e1011060. doi: 10.1371/journal.pgen.1011060 (PMC10727445; doi:10.1371/journal.pgen.1011060)
Supplement: S5 Fig — a) Sequence logo of enriched motif in σH- dependent genes identified using RNAseq (downregulated >3-fold; padj < 0.01) using MEME Suite 5.5.1. b) Location of conserved motif in upstream regions of σH- dependent genes. Fold downregulation of each genes in ΔsigH strain is also noted. Difference in base composition of nucleotide immediately downstream to conserved GGAA motif is indicated in green (G/C) and yellow (A/T). c) Possible hierarchy within the σH regulon. (PDF) [file pgen.1011060.s009.pdf]

a

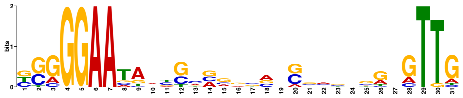

b

Gene	Product		Wt-Tet/ΔsigH-Tet
MAB_1528c	Probable reductase	gcggctgc ccgGGAATactcgacgg--cgcctcgtGTTG ggttgacc	23.5
MAB_3016c	putative oxidoreductase	gtaggcgg cggGGAATatcgcggg-cgatcgaataaTTG gactgcca	12.5
MAB_4143c	Putative anti-sigma	agcccaca ggaGGAATgcacagaccgtcaaacgggGTTG ctctggcc	17
MAB_0827	fabG like reductase	tggtgtga cggGGAATgtttcggac-cccagtccgcttG aaagtcat	10.5
MAB_2739c	probable trxB	tgaccgcc ggcGGAACatcgcgca-ctccggcaacGTTG aggactac	9.6
MAB_0485	putative oxidoreductase	tgaccagc cggGGAAGcatcccgg-cgatatctgcGTTG gactgtgc	7.5
MAB_3461c	probable oxidoreductase	ttgcgccg ccgGGAATaatgtcag-ctcgattgacATTa tggtgatt	4.6
MAB_3543	SigH	gagatact ttgGGAAAtaccccggcatgcgtcggtGTTG agaagtct	4.2
MAB_2886c	hypothetical protein	aatcaccg ccGGGAATcaaacgtcac-tagacggcGTTa ctgtgatg	4.0
MAB_4663	hypothetical protein	acagtgga ggcGGAAAtacccactaatggatacacGTTG ccgtaagc	3.6
MAB_4748c	pyridoxaminePO_4oxidase	aggagtgg gggGGAATaaggggtgcggggccgatgGTTt gatgccac	3.4
MAB_4664	hypothetical protein	gaggcatc gcaGGAAGatcttaaaagtattggagcacag acaaaatg	3.6
MAB_1339	Probable oxidoreductase	cacccgga gcgGGAATagcgaaccgacggcggcggGTTG gccgagac	3.3
MAB_1362	SigE	ccgcattc tcaGGAAGagttaagtta-gctcctaaGTgG cccacacc	3.2
MAB_3639	probable oxidoreductase	cgtcccac gggGGAATagcggaga-aacaccaggaGTTG cggtaaat	2.8
MAB_1572	dsbA-like oxidoreductase	gaaccgcg tcgGGAACagggcggc-acgatggggaGTTG cgttactt	2.6

c

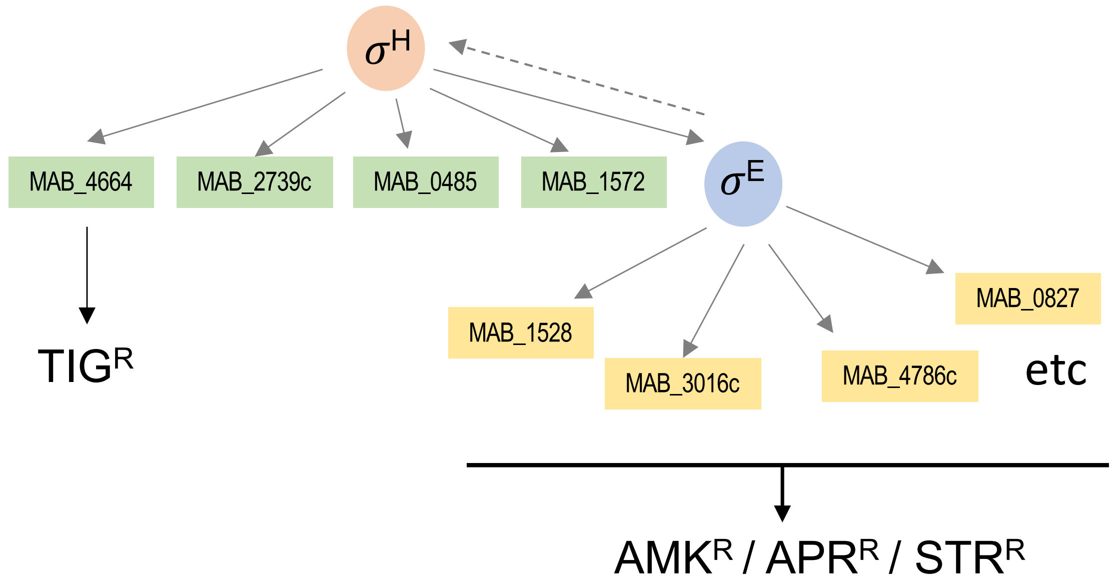

Figure S5
